# Combining loss of function of *FOLYLPOLYGLUTAMATE SYNTHETASE1* and *CAFFEOYL*-*COA 3*-*O*-*METHYLTRANSFERASE1* for lignin reduction and improved saccharification efficiency in *Arabidopsis thaliana*

**DOI:** 10.1186/s13068-019-1446-3

**Published:** 2019-05-03

**Authors:** Hongli Xie, Nancy L. Engle, Sivasankari Venketachalam, Chang Geun Yoo, Jaime Barros, Mitch Lecoultre, Nikki Howard, Guifen Li, Liang Sun, Avinash C. Srivastava, Sivakumar Pattathil, Yunqiao Pu, Michael G. Hahn, Arthur J. Ragauskas, Richard S. Nelson, Richard A. Dixon, Timothy J. Tschaplinski, Elison B. Blancaflor, Yuhong Tang

**Affiliations:** 1 Noble Research Institute, LLC, 2510 Sam Noble Parkway, Ardmore, OK 73401 USA; 2 Biosciences Division, Oak Ridge National Laboratory, Oak Ridge, TN 37831 USA; 3 Complex Carbohydrate Research Center, University of Georgia, 315 Riverbend Road, Athens, GA 30602 USA; 4 BioDiscovery Institute and Department of Biological Sciences, University of North Texas, Denton, TX 76203 USA; 5 BioEnergy Science Center, United States Department of Energy, Oak Ridge, TN 37831 USA; 6 The Center for Bioenergy Innovation, United States Department of Energy, Oak Ridge, TN 37831 USA

**Keywords:** *folylpolyglutamate synthetase1*, *fpgs1*, *caffeoyl*-*CoA 3*-*O*-*methyltransferase1*, *ccoaomt1*, Glycome profiling, Metabolite profiling, Phenylpropanoid pathway, Lignin

## Abstract

**Background:**

Downregulation of genes involved in lignin biosynthesis and related biochemical pathways has been used as a strategy to improve biofuel production. Plant C1 metabolism provides the methyl units used for the methylation reactions carried out by two methyltransferases in the lignin biosynthetic pathway: caffeic acid 3-*O*-methyltransferase (COMT) and caffeoyl-CoA 3-*O*-methyltransferase (CCoAOMT). Mutations in these genes resulted in lower lignin levels and altered lignin compositions. Reduced lignin levels can also be achieved by mutations in the C1 pathway gene, *folylpolyglutamate synthetase1* (*FPGS1*), in both monocotyledons and dicotyledons, indicating a link between the C1 and lignin biosynthetic pathways. To test if lignin content can be further reduced by combining genetic mutations in C1 metabolism and the lignin biosynthetic pathway, *fpgs1ccoaomt1* double mutants were generated and functionally characterized.

**Results:**

Double *fpgs1ccoaomt1* mutants had lower thioacidolysis lignin monomer yield and acetyl bromide lignin content than the *ccoaomt1* or *fpgs1* mutants and the plants themselves displayed no obvious long-term negative growth phenotypes. Moreover, extracts from the double mutants had dramatically improved enzymatic polysaccharide hydrolysis efficiencies than the single mutants: 15.1% and 20.7% higher than *ccoaomt1* and *fpgs1*, respectively. The reduced lignin and improved sugar release of *fpgs1ccoaomt1* was coupled with changes in cell-wall composition, metabolite profiles, and changes in expression of genes involved in cell-wall and lignin biosynthesis.

**Conclusion:**

Our observations demonstrate that additional reduction in lignin content and improved sugar release can be achieved by simultaneous downregulation of a gene in the C1 (*FPGS1*) and lignin biosynthetic (*CCOAOMT*) pathways. These improvements in sugar accessibility were achieved without introducing unwanted long-term plant growth and developmental defects.

**Electronic supplementary material:**

The online version of this article (10.1186/s13068-019-1446-3) contains supplementary material, which is available to authorized users.

## Background

Lignocellulosic biomass is viewed as a desirable sustainable feedstock for the production of biofuels because of its abundance and renewable nature [1–4]. Lignin, a major component of lignocellulose, cross-links with hemicellulose in the cell wall to form the lignin–hemicellulose matrix [5–8]. The lignin and hemicellulose matrix encrusts the cellulose skeleton, providing mechanical support for the growing plant. Moreover, the lignocellulose complex forms a barrier allowing unimpeded substrate transport protecting plants against pathogens, which are critical for plant growth, development, and survival [9, 10]. The amount and composition of lignin differs among species and individual tissues, as well as in tissues under different plant growth conditions or developmental stages. To produce biofuel from lignocellulosic biomass, polysaccharides must be separated or loosened from the lignocellulose matrix for hydrolyzation into sugar subunits and fermentation into ethanol. However, the presence of lignin reduces hydrolyzation rates (e.g., saccharification efficiency) by limiting enzyme access to the polysaccharides. The reduction of lignin content or modification of its structure is important not only for biofuel applications, but also for improving the digestibility of some plants for animal consumption [11–16].

Lignin biosynthesis consists of three steps: the synthesis of lignin monolignols, their transport to the lignifying sites, and the polymerization of monolignols into the growing lignin polymer [9]. Monolignol biosynthesis starts with the amino acid, l-phenylalanine, and proceeds through the general phenylpropanoid pathway [13, 17–20]. There are three basic types of monomer units: the *p*-hydroxyphenyl (H), guaiacyl (G), and syringyl (S) units. Besides hydroxylation, the major difference between the three lignin monomers is the degree of methylation on the aromatic ring. In plants, there are two *O*-methyltransferases in the lignin biosynthesis pathway, namely, caffeoyl coenzyme A 3-*O*-methyltransferase (CCoAOMT) and caffeoyl-CoA 3-*O*-methyltransferase (COMT). COMT is responsible for the *O*-methylation at the C5 position of the phenolic ring while CCoAOMT’s function is in the *O*-methylation of the C3 position [21, 22]. However, there are reports that COMT is also involved in the *O*-methylation at the C3 position [22, 23].

CCoAOMT was first implicated in lignin production during studies showing the differentiation of tracheary elements in cultured Zinnia mesophyll cells [24]. It is now well understood that CCoAOMT is the key methylation enzyme for caffeoyl-CoA, producing feruloyl-CoA, an upstream component of the G and S monolignol branches [22, 25, 26]. Mutation or transgenic downregulation of *CCoAOMT* genes causes changes in lignin content and composition in a number of plant species such as tobacco (*Nicotiana tabacum*) [25–27], woody poplar (*Populus tremula* x *Populus alba*) [21, 28], alfalfa (*Medicago sativa*) [29–31], flax (*Linum usitatissimum*) [32], pine (*Pinus radiata*) [33], maize (*Zea mays*) [34], and thale cress (*Arabidopsis thaliana*) [22, 35]. There are seven putative *CCoAOMTs* in the Arabidopsis genome [36], with *CCoAOMT1* [*At4g34050*] confirmed to be the primary family member involved in the monolignol pathway [22].

In plants, synthesis of major end products such as lignin, phytohormones, betaines, and alkaloids requires methylation reactions [37–39]. The required methyl groups are donated by the universal methyl group donor *S*-adenosylmethionine (AdoMet), and they originate from the C1 derivatives of the cofactor tetrahydrofolate. Lignin accumulation in plants was thus predictably affected by mutations in genes responsible for producing AdoMet, or in genes responsible for maintaining pools of the folate C1 derivatives. Mutation of *S*-*adenosylmethionine synthetase3* (*SAMS3*), whose protein product catalyzes synthesis of AdoMet from l-methionine and ATP, leads to over-accumulation of methionine and a significant decrease in total lignin content in Arabidopsis [40]. Folates play a central role in C1 metabolism by providing one-carbon groups for methylation reactions in living organisms. In maize, the *brown*-*midrib* (*bm*) natural mutants, *bm2* and *bm4*, are disrupted in genes encoding a methylenetetrahydrofolate reductase (MTHFR) [41] and a folylpolyglutamate synthase (FPGS) [42], respectively. Reductions in lignin content and altered lignin composition were observed in both *bm* mutants [41, 42]. We recently reported that mutation of *FPGS1* by T-DNA insertions in Arabidopsis resulted in lower lignin content and reduced cell-wall recalcitrance [43].

One method to improve plants for biofuel production while maintaining their normal growth is to alter the expression of multiple genes in the lignin biosynthesis pathway as opposed to one. Three double-mutant combinations of *peroxidase* (*Prx*) among *AtPrx2*, *AtPrx25,* and *AtPrx71* in Arabidopsis had near normal growth phenotypes with reduced lignin contents [44]. Similarly, Arabidopsis double mutants in which the expression of *transaldolase* (*TRA2*), *cinnamate 4*-*hydroxylase* (*C4H*), or *4*-*coumarate:CoA ligase* (*4CL1*) with a *COMT* gene were simultaneously reduced, resulted in higher saccharification efficiency without compromising plant biomass yield [45]. Transgenic tobacco plants in which *cinnamyl alcohol dehydrogenase* (*CAD*) and *cinnamoyl CoA reductases* (*CCR*) were simultaneously downregulated, also had reduced lignin and normal growth [46]. However, some double mutants or transgenetically altered plants targeting two genes for downregulation, such as Arabidopsis *ccoaomt1 comt1* and switchgrass hydroxycinnamoyl CoA:shikimate hydroxycinnamoyl transferase (HCT) 1 and 2, have compromised growth [22, 47], suggesting that not all mutant combinations of genes in lignin biosynthesis present practical strategies for overcoming recalcitrance. One further modification that has succeeded in returning plant growth to normal was to downregulate or overexpress other lignin-related genes or transcription factors [48–50]. Such elaborate experimental approaches to modify lignin content enable the plants with reduced lignin content not only for practical biofuel-based applications but also as tools for gaining a deeper understanding of regulatory mechanisms underlying lignin biosynthesis.

To explore other possibilities for lignin reduction through multiple gene downregulations and to further understand the interaction of lignin biosynthesis with the C1 metabolic pathway, double mutants between *fpgs1* and *ccoaomt1* were generated in Arabidopsis, and their growth phenotype and cell-wall biochemistry/recalcitrance were studied. Our results show that simultaneous downregulation of a lignin biosynthetic gene and a C1 metabolic gene alters lignin composition and increases sugar release in Arabidopsis without long-term adverse growth impacts.

## Results

### Expression patterns of *CCoAOMT1* and *FPGS1* in Arabidopsis stems

**Fig. 1:**
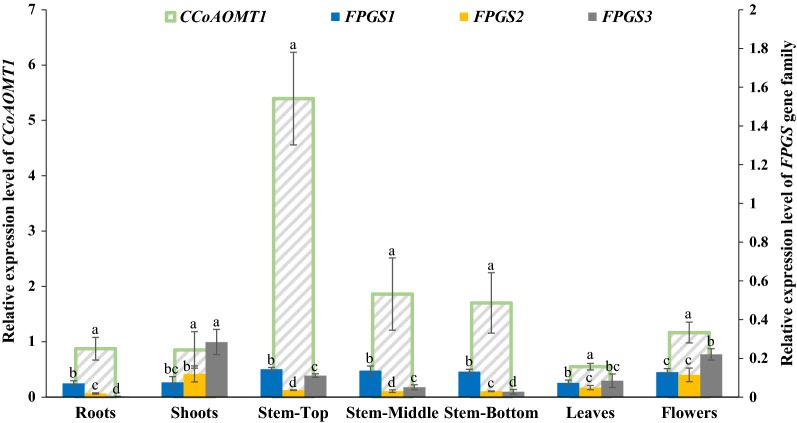
Expression analysis of *CCoAOMT1* and *FPGS* gene family members in different organs of wild-type (WT) Arabidopsis plants by qRT-PCR. Relative transcript levels of *CCoAOMT1* are presented on the left y axis and *FPGS* gene family are presented on the right y axis. The labels for both sides are of different scales. Root and shoot tissues were from 2-week-old seedlings; Stem, leaf (youngest leaves), and flower (opened flowers) tissues were from 6-week-old plants; Stem-Top: top parts of stem above 1st silique; Stem-Middle: stem tissues between 1st branch and 1st silique; Stem-Bottom: stem tissues below the 1st branch; The *AtACTIN2* (*AT3G18780*) was used as the reference gene. Relative fold changes were obtained by qRT-PCR using the 2^−ΔΔCT^ method. Values are means ± SD from three biological replicates with three technical replicates per biological replicate. Each biological replicate contained 20 plants. Different letters indicate statistically significant differences between values according to one-way ANOVA and LSD test (*P* ≤ 0.05)

In corn, FPGS and CCoAOMT are important for lignin production, as shown by the reduced lignin content observed for plants mutated or downregulated for *FPGS* [42] or *CCoAOMT* expression [34]. *FPGS1* and *CCoAOMT1* are highly expressed in lignified Arabidopsis stems [22, 43]. To study their interactions in relation to lignin biosynthesis, the expression profiles of *CCoAOMT1* and the *FPGS* gene family were examined under the same growth conditions. The samples used for transcript level analysis included root and shoot tissues from 2-week-old Arabidopsis (col-0) plants as well as leaf, flower, and stem tissues from 6-week-old Arabidopsis (col-0) plants. Quantitative RT-PCR results showed that the across-all-tissues expression levels of *CCoAOMT1* was higher than the expression levels of the *FPGS* gene family. The high transcript level of *CCoAOMT1* in the stems, especially in the top portion of 6-week-old stem where lignification is occurring, demonstrates the correlation between CCoAOMT1 activity and lignification (Fig. 1). Among members of the *FPGS* gene family, *FPGS1* had a relatively high expression level in different portions of the stem and flower tissues of 6-week-old plants (Fig. 1). *FPGS2* and *FPGS3* had relatively high expression levels in shoots of 2-week-old plants and flowers of 6-week-old plants (Fig. 1). The expression level of *FPGS1* is the highest among three gene family members in the stem, indicating that it is the best isoform to study interactions between C1 and lignin biosynthesis pathways in Arabidopsis stems.

### Generation and characterization of single and double mutants

**Fig. 2:**
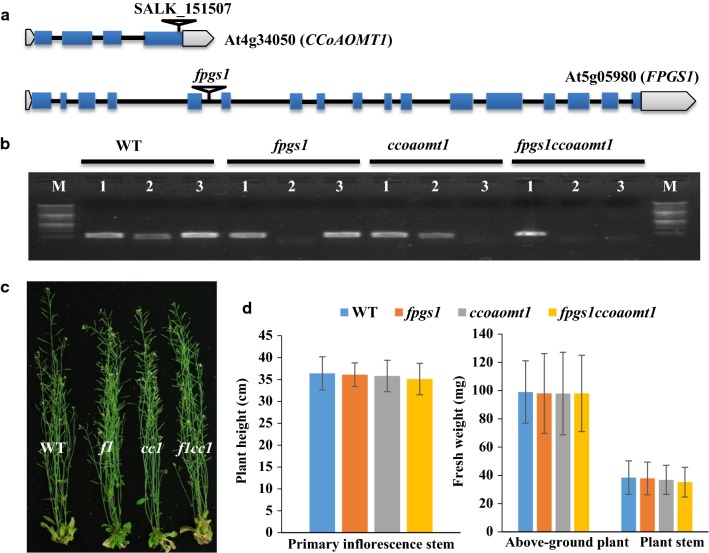
Characterization of Arabidopsis *fpgs1, ccoaomt1, and fpgs1ccoaomt1* mutants. **a** Schematic diagram of the exon–intron organization of *CCoAOMT1* and *FPGS1* genes, and the T-DNA insertion positions in *ccoaomt1* (SALK_151507) and *fpgs1* mutants. **b** Confirmation of *FPGS1* and *CCoAOMT1* transcript levels in 9-day-old plate-grown WT, *fpgs1*, *ccoaomt1*and *fpgs1ccoaomt1* by RT-PCR (Lane M, 1 kb DNA ladder Promega G5711; lane 1, housekeeping control *AtACTIN 2* gene; lane 2*, FPGS1* gene; lane 3, *CCoAOMT1* gene). **c** 6-week-old WT, *fpgs1*(*f1*), *ccoaomt1* (*cc1*), and *fpgs1ccoaomt1* (*f1cc1*) Arabidopsis plants prior sampling the stems for analysis (*n* ≥ 30). **d** Comparisons of plant height, fresh stem weight, and fresh aboveground weight of 6-week-old WT, *fpgs1*, *ccoaomt1,* and *fpgs1ccoaomt1* Arabidopsis plants. Plant height is the height of the primary inflorescence stem; Aboveground plant fresh weight: include all aboveground tissues including leaves, flowers, and siliques; Plant stem fresh weight: aboveground tissue after removal of rosette leaves, cauline leaves, flowers, and siliques. The data were collected from 30 plants for each genotype. There were no statistically significant differences between values according to one-way ANOVA and LSD test (values were mean ± SE. *n* = 30, *P* ≤ 0.05)

The *fpgs1* Arabidopsis mutant used in this study was the *fpgs1*-*1* line [43]. The DNA sequence in the region of *FPGS1* in the specific homozygous *fpgs1* plant used to cross with the *ccoaomt1* mutant was confirmed by PCR using genotyping primers (Additional file 1: Table S1). The T-DNA insertion was confirmed to be in the fifth intron of gene *FPGS1* (Fig. 2a) as previously described [51]. The homozygous *fpgs1* plants did not show obvious growth defects compared to the wild type except for the short root phenotype in the seedling stage (Additional file 2: Fig. S1).

The DNA sequence in the region of the homozygous *ccoaomt1* plant used to cross with the *fpgs1* plant was determined by PCR using genotyping primers and coincided with the sequence determined for SALK_151507 (Additional file 1: Table S1). There were two contiguous T-DNAs inserted in tandem in the fourth exon of the *CCoAOMT1* gene (Fig. 2a). The homozygous *ccoaomt1* plants, similar to the *ccoaomt1*-*3* line previously reported [23], had no significant growth defects under our growth conditions.

The homozygous *fpgs1* (♀) and *ccoaomt1* (♂) Arabidopsis mutants were crossed to generate F1 plants whose F2 plants would segregate. Homozygous single and double mutants were obtained from the F2 population and confirmed by PCR using genotyping primers (Additional file 1: Table S1). We also investigated *FPGS1* and *CCOAOMT1* expressions in single and double homozygous mutants. The RT-PCR results showed that expression level of both genes were beyond detection in corresponding single and double homozygous mutants 9 days after germination (Fig. 2b).

The growth and development of single and double homozygous mutants were examined. Under long-day conditions, aerial growth of the double-mutant *fpgs1ccoaomt1*, was visually similar to the single mutants and wild type (WT) 6 weeks post germination (Fig. 2c). Aerial phenotypic traits, including plant height, fresh weight of aboveground plant material, and stem material, were compared between WT, single, and double mutants from 6-week-old plants (Fig. 2d). The results showed that the measured growth traits for the double-mutant *fpgs1ccoaomt1* were comparable to WT and each of the single mutants except for root length at early stages of growth, in which it was shorter than WT and *ccoaomt1* but similar to the *fpgs1* mutant (Additional file 2: Fig. S1).

### Impact of *FPGS1* and *CCoAOMT1* disruption on lignin content and composition in the *fpgs1ccoaomt1* mutant

**Fig. 3:**
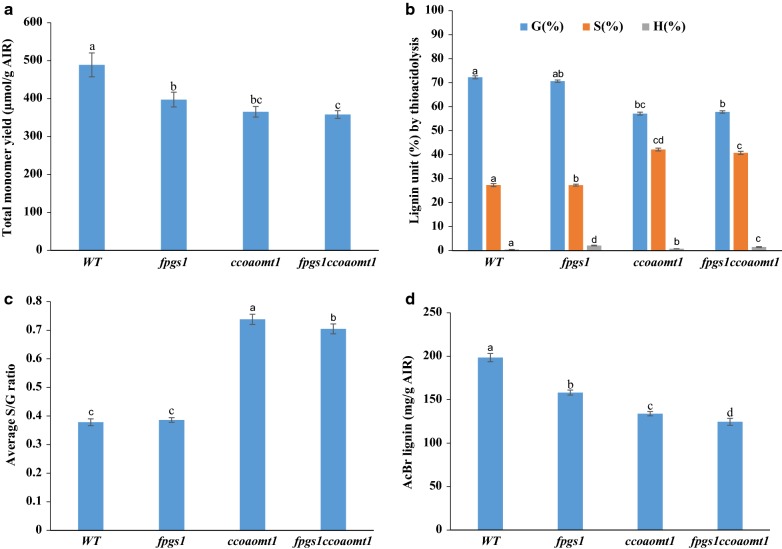
Lignin composition and acetyl bromide (AcBr) lignin content analysis in 6-week-old Arabidopsis stems from WT, *fpgs1*, *ccoaomt1*, and *fpgs1ccoaomt1* plants. **a** Total lignin monomer yield (μmol/g AIR) determined with thioacidolysis. **b** Lignin monomer percentage calculated by total thioacidolysis yield. **c** S/G ratio of lignin monomers obtained by thioacidolysis analysis. **d** Total AcBr lignin content (mg/g AIR). Different letters in column indicate statistically significant differences between values according to one-way ANOVA and LSD test (values were mean ± SE. *n* = 3, *P* ≤ 0.05. Each biological replicate included mature inflorescence stems pooled from 20 individual plants)

GC-coupled thioacidolysis was used to measure the relative content of lignin monomers bound by β-*O*-4 linkages [52] in the stems of 6-week-old double-mutant *fpgs1ccoaomt1,* single mutants *fpgs1* and *ccoaomt1,* and WT plant tissue. When monomer subunits were totaled, stems from the single mutants, *fpgs1* and *ccoaomt1,* and the double-mutant, *fpgs1ccoaomt1*, showed decreases, respectively, of 18.7%, 25.2%, and 26.7%, compared with WT stems (Fig. 3a). Relative content for each monomer was calculated from thioacidolysis-derived lignin monomers for each genotype (Fig. 3b). When compared with WT stems, the *fpgs1* stems had slight reduction of G- and S- monomers but had a significant 3.6-fold increase of H- monomers (Fig. 3b). The *ccoaomt1* stems had a 21% reduction of G-monomers and a 54% increase of S-monomers while had a slight increase (7%) of H-monomers (Fig. 3b). The double-mutant stems had a 20% reduction of G-monomers while a 49% increase of S-monomers and a significant 3.6-fold increase of H-monomers (Fig. 3b). Based on thioacidolysis, there was no change of S/G ratio in *fpgs1* tissue compared with WT tissue. On the contrary, there were very significant increases in S/G ratios in both the double mutant and *ccoaomt1* compared with WT plants (Fig. 3c). The higher S/G ratio in Arabidopsis *ccoaomt1* mutants was also reported in other studies [22, 35]. Based on two-dimensional (2D) ^1^H–^13^C heteronuclear single-quantum coherence (HSQC) NMR, *fpgs1* tissue had relatively more H units than WT tissue, similar to what was observed by thioacidolysis, while maintaining the same S/G ratio as WT tissue (Additional file 3: Table S2). Similar to what was measured by thioacidolysis, *ccoaomt1* and *fpgs1ccoaomt1* showed decrease of relative content of G units, and relative increase of S and H units; the S/G ratios in *ccoaomt1* and *fpgs1ccoaomt1* were significantly increased compared with WT tissue (Additional file 3: Table S2).

Total lignin content in stems of 6-week-old plants was also determined by the acetyl bromide (AcBr) method [52], which, unlike thioacidolysis, is independent of linkage composition. The single mutants *fpgs1* and *ccoaomt1* had 20% and 32.5% reductions of AcBr lignin, respectively, compared with the WT plants (Fig. 3d). The double mutant had a 37.2% reduction of AcBr lignin compared with the WT plants (Fig. 3d), indicating a further reduction of lignin content in *fpgs1ccoaomt1* compared with each of the single mutants. A 17% reduction in AcBr lignin content was also observed previously in the single Arabidopsis mutant *fpgs1* [43]. Reduced AcBr lignin content and an increase of the S/G ratio in *ccoaomt1* mutants were also reported in earlier work in Arabidopsis [22, 35].

In addition to total monomer yield, the linkage pattern affects the property of lignins, e.g., the percentage of β-*O*-4-ether bonds can closely reflect the degree of condensation of the lignin polymer in the samples and potentially affect plant recalcitrance [53]. 2D HSQC-NMR was also used to analyze the relative abundance of lignin interunit linkages. The distribution patterns of the main interunit linkages in *fpgs1* were *s*imilar to those in the wild type. Both *ccoaomt1* and *fpgs1ccoaomt1* showed slightly higher proportions of β-*O*-4 linkages, and lower numbers of β-5 linkages than WT-*fpgs1* (Additional file 3: Table S2). These results indicated that the distribution of interunit linkages and lignin composition in the double-mutant *fpgs1ccoaomt1* were more similar to those in *ccoaomt1* than to *fpgs1*. Furthermore, two functional groups, the acetyl and the methoxyl, were measured by NMR analysis. All mutants had higher levels of acetyl groups than in the WT controls, and there was no difference among the mutants. Similar levels of methoxyl groups were observed for all three mutants and WT control (Additional file 3: Table S2).

### Saccharification yield and enzymatic hydrolysis efficiency is increased in the *fpgs1ccoaomt1* mutant

Total sugar and enzymatically released sugar were determined for the stems of 6-week-old double-mutant, *fpgs1ccoaomt1,* each single-mutant, and wild-type Arabidopsis plants through the phenol–sulfuric acid method (PSA) [54]. Based on PSA, total sugar release from destarched alcohol-insoluble residue (AIR) without acid pretreatment was 10.5%, 14.2%, and 16.9% more in the *fpgs1*, *ccoaomt1,* and *fpgs1ccoaomt1* extracts, respectively, compared with WT extracts (Table 1).

**Table 1:** Total sugar release, enzymatic sugar release, and enzymatic hydrolysis efficiencies in 6-week-old stems of WT*, fpgs1*, *ccoaomt1*, and *fpgs1ccoaomt1* plants

Line name	Total sugar release (mg/g AIR)	Enzymatic sugar release (mg/g AIR)	Enzymatic hydrolysis efficiencies (%)
WT	530.1 ± 29.1^c^	122.5 ± 15.5^c^	21.8 ± 2.6^c^
*fpgs1*	585.9 ± 26.7^b^	136.6 ± 9.1^bc^	22.9 ± 3.2^bc^
*ccoaomt1*	605.3 ± 11.5^ab^	145.1 ± 26.4^b^	24.0 ± 3.5^b^
*fpgs1ccoaomt1*	619.7 ± 21.0^a^	169.9 ± 17.5^a^	27.7 ± 4.1^a^

Total sugar release (TSR) and enzymatic sugar release (ESR) were estimated from destarched alcohol-insoluble residues (AIR) of stem tissues from 6-week-old Arabidopsis plants without acid pretreatment. Enzymatic hydrolysis efficiency is expressed as a percentage of the ESR yield to the TSR yield for each replicate prior to obtain average values. Each value represents six biological replicates and each biological replicate contained 20 plants. Different letters in each column indicate statistically significant differences between values in the columns according to one-way ANOVA and LSD test (values are mean ± SE, *n* = 6, *P* ≤ 0.05)

Enzymatically released sugar from destarched AIR, without acid pretreatment, was 11.5%, 18.5%, and 38.7% more in *fpgs1*, *ccoaomt1*, and the double mutant than WT stems, respectively (Table 1). When compared to each single mutant, the double mutant had 24.4% and 17.0% higher enzymatically released sugar than *fpgs1* and *ccoaomt1* extracts, respectively (Table 1). Subsequently, the enzymatic hydrolysis efficiency was calculated as the percentage of enzymatic sugar release yield to the total sugar release yield [30]. When compared to WT, *fpgs1*, *ccoaomt1*, and the double-mutant extracts showed a 5.0%, 10.5%, and 27.2% increase in enzymatic hydrolysis efficiency, respectively (Table 1). When compared to each single mutant, the double mutant had a 20.7% increase versus the *fpgs1* value and a 15.1% increase versus the *ccoaomt1* value, for its enzymatic hydrolysis efficiency (Table 1).

Overall the simultaneous disruption of *FPGS1* and *CCOAOMT1* expression in plants increased the total sugar release, enzymatic sugar release, and enzymatic hydrolysis efficiency compared with disruption of each gene alone.

### Glycome profiling reveals major differences in non-cellulosic cell-wall components between mutant and WT plants

**Fig. 4:**
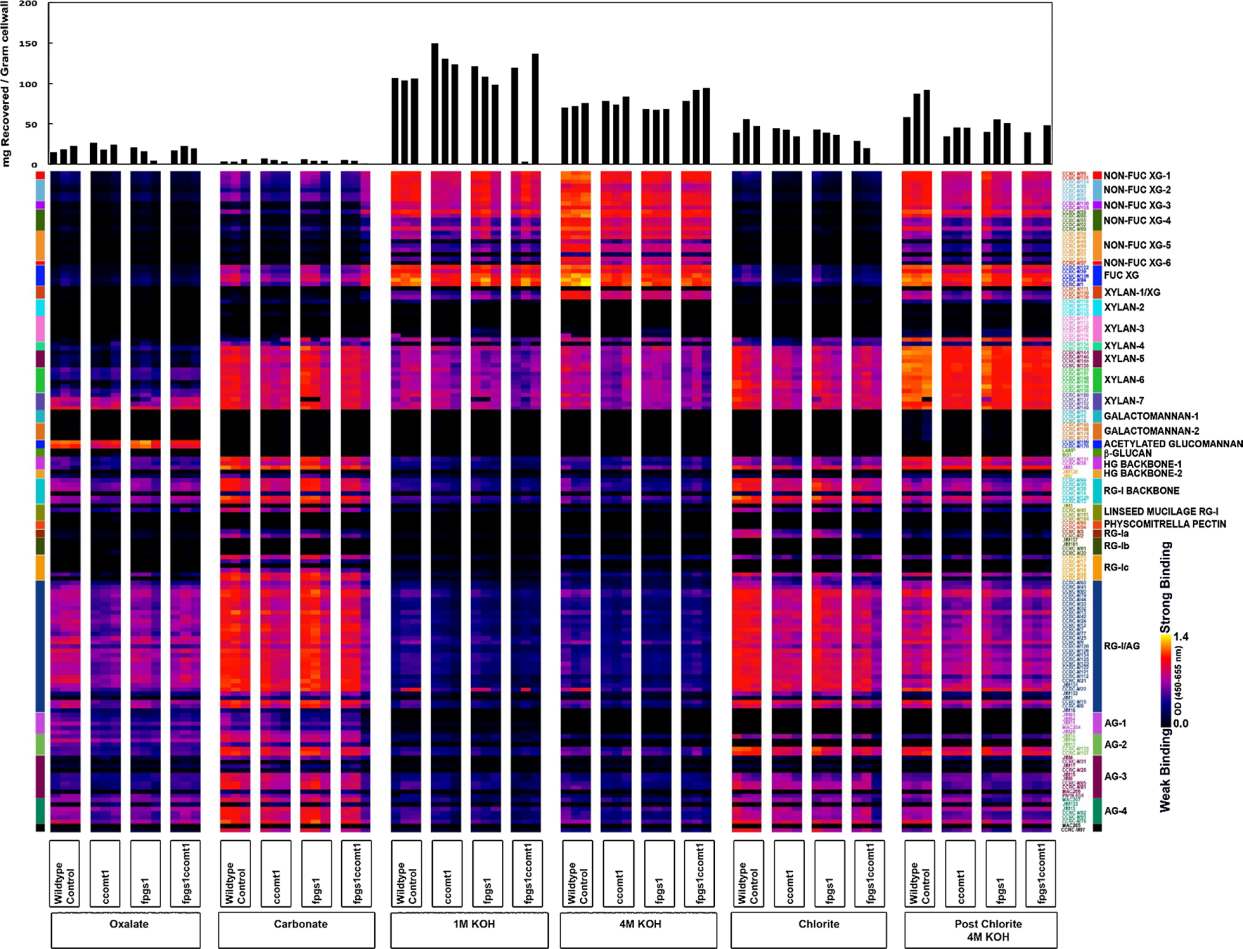
Glycome-profiling heatmaps of sequential extracts by increasingly harsher reagents from alcohol-insoluble (cell wall) residues (AIR) prepared from 6-week-old Arabidopsis stems of WT*, fpgs1*, *ccoaomt1*, and *fpgs1ccoaomt1* plants. The top bar graph showed the total carbohydrates extracted from each reagent (from ammonium oxalate, sodium carbonate, 1 M KOH, 4 M KOH, acidic sodium chlorite, to 4 M KOH PC). The heatmap indicated the strength of different monoclonal antibodies (mAbs) binding with the non-cellulosic cell-wall components extracted from each reagent. Bright yellow showed the strongest binding, dark blue for no binding

The AIR materials from 6-week-old stems for all four genotypes, WT, *fpgs1*, *ccoaomt1*, and the double mutant, were used for glycome profiling. Glycome profiles can show the extractability of various non-cellulosic cell-wall components, i.e., xyloglucan, xylan, and pectin/arabinogalactan epitopes, through six sequential extractions under increasingly harsh conditions. When considering total non-cellulosic carbohydrate released from AIR during each of the extractions, there were more carbohydrates extracted from mutants at moderate harshness. For example, under 1 M KOH condition, there were more carbohydrates recovered for all three mutants; there were a noticeable increase in the amount of total carbohydrates extracted for double mutants in 4 M KOH. The extractability of non-cellulosic carbohydrate reversed in the two most harsh conditions, with the mutants having less non-cellulosic carbohydrates recovered while the wild type had more. For example, the double mutant had the least recovered carbohydrates under Chlorite extraction condition while the wild type had the most under the Post Chlorite 4 M KOH extract condition, among all four genotypes (Fig. 4). These results indicated that the extractability of non-cellulosic carbohydrates from all mutants’ cell wall was higher than those from wild-type cell walls. Most noticeable is the highest extractability of non-cellulosic carbohydrates from cell wall of double-mutant *fpgs1ccoaomt1* (Fig. 4).

When equal amount of carbohydrates extracts for each extract was loaded for epitope detection, the glycome-profiling results showed high resemblance of each mutant to those of wild-type lines. A limited number of carbohydrates differed between the mutants when each was compared against WT. Only very few epitopes show significant difference among the genotypes. When comparing glycome-profiling data of each single mutant against that of the double mutant, the difference between *fpgs1* and *ccoamt1* was minor but clear. In *fpgs1*, there were 19 glycan epitopes of higher levels and four of lower levels, compared with the double mutant. By contrast, there were 15 glycan epitopes with lower levels and only three with higher levels in *ccoamt1,* compared with the double mutant (Additional file 4: Fig S2). Very few selected epitopes showed similar trends between *fpgs1* and *ccoaomt1* when each was compared against the double mutant. These changes indicated subtle structure changes in the non-cellulosic components of cell wall among WT plants and the single and double *fpgs1 ccoamt1* mutants.

### Metabolic profiling displayed shifts in phenylpropanoid pathway in *fpgs1*, *ccoaomt1,* and *fpgs1ccoaomt1* mutants

**Fig. 5:**
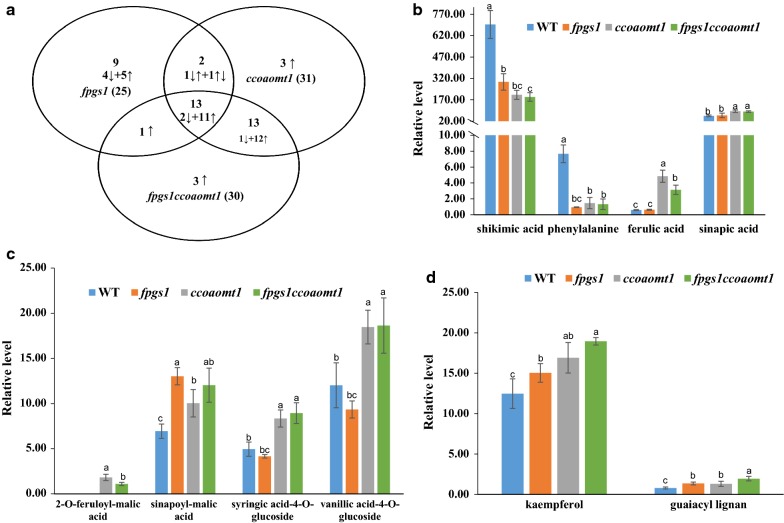
Soluble metabolites analysis by GC/MS of stem extracts from 6-week-old WT, *fpgs1*, *ccoaomt1*, and *fpgs1ccoaomt1* plants. **a** A Venn diagram presentation of the up- and down- regulated compounds significantly altered in *fpgs1*, *ccoaomt1,* and *fpgs1ccoaomt1* compared with WT. **b** The relative content (ug/g fresh weight; sorbitol equivalents) of shikimic acid, phenylalanine, ferulic acid, and sinapic acid identified by GC–MS among four genotypes. **c** The relative content (ug/g fresh weight; sorbitol equivalents) of sinapoyl-malic acid, 2-*O*-feruloyl-malic acid, syringic acid-4-*O*-glucoside, and vanillic acid-4-*O*-glucoside among four genotypes. d. The relative content (ug/g fresh weight; sorbitol equivalents) of kaempferol and guaiacyl lignan identified among four genotypes. Different letters between values show statistically significant differences according to one-way ANOVA and LSD test (Values are mean ± SE. *n* = 4, *P* ≤ 0.05. Each biological replicate included mature inflorescence stems pooled from 20 individual plants). A more detailed illustration of the differentially accumulated metabolites is shown in Additional file 6: Table S5

Metabolite analysis of 6-week-old Arabidopsis stems was performed by gas chromatography mass spectrometry (GC–MS) to determine the impact on metabolism after the disruption of *FPGS1*, *CCOAOMT1*, or *FPGS1/CCOAOMT1* combined expression relative to non-disrupted expression in WT stems. More than 100 peaks were detected in the stem tissues of all four genotypes. The top 100 peaks were chosen for analysis, and 77 of them were of known chemical structures (Additional file 5: Fig. S3). Based on *P* < 0.05, levels for 25, 31, and 30 metabolites were significantly different from WT for *fpgs1, ccoaomt1*, and the double-mutant, *fpgs1ccoaomt1*, respectively. Comparing the levels of detected compounds among all genotypes revealed that the metabolite profile for the *fpgs1ccoaomt1* double mutant was more similar to *ccoaomt1* than to *fpgs1* (Fig. 5a; Additional file 5: Fig S3).

Levels of nine compounds were uniquely changed in the *fpgs1* mutant versus WT stem tissue. Among them, four sugars were decreased: glucose, fructose, 3,6-anhydrogalactose, and galactose (Fig. 5a, Additional file 6: Table S3). Levels of three compounds—maleic acid, glutamic acid, and sucrose—were uniquely increased in *ccoaomt1* (Fig. 5a, Additional file 6: Table S3). Levels for 13 compounds exhibited similar alterations in all three mutants compared with WT, with 11 increased and two decreases (Fig. 5a). Phenylalanine and shikimic acid were among the compounds with significant decreases (Fig. 5b). Among the compounds that were increased in levels in all three mutants, guaiacyl lignan and sinapoyl-malic acid are directly related to the phenylpropanoid pathway (Fig. 5c and d, Additional file 6: Table S3).

For another 13 compounds, levels for 12 were increased and one (threonic acid) decreased similarly in *ccoaomt1* and double mutants compared with values from WT tissue. Levels of these compounds were unchanged in *fpgs1* mutant tissue compared with WT tissue. A significant number of metabolites related to the phenylpropanoid/flavonoid pathways are among this group of metabolites which increased in the *ccoaomt1* and double-mutant tissue: ferulic acid, 2-*O*-feruloyl-malic acid, and 16.25 398 383 glucoside, increased fivefold or more; two flavonoid compounds, syringic acid-4-*O*-glucoside and vanillic acid-4-*O*-glucoside, increased approximately twofold; sinapic acid, a precursor of syringyl lignin and bioactive compound within the cell walls, increased one-third and an increase in compound 2-*O*-feruloyl-malic acid was only detectable in *ccoaomt1* and the double mutant among four genotypes (Fig. 5b, c; Additional file 6: Table S3). There were only two metabolites of levels that increased in the double-mutant tissue compared with each single-mutant tissue, and they were guaiacyl lignan and kaempferol (Fig. 5d; Additional file 6: Table S3), which are direct products of the phenylpropanoid pathway.

### Transcript profiling of *fpgs1ccoaomt1* mutant tissue reveals molecular impacts of lignin biosynthesis pathway by *FPGS1* and *CCoAOMT1*

Transcriptomes of single and double mutants of *fpgs1* and *ccoaomt1* were investigated in 6-week-old Arabidopsis stems. A total of 392 differentially expressed genes were identified among *fpgs1, ccoaomt1*, and the double mutant compared with WT. There were 161, 250, and 234 upregulated and 13, 22, and 25 downregulated genes in *fpgs1, ccoaomt1,* and the double-mutant stem tissue, respectively, when compared with WT stem tissue. The quality of the microarray data was supported by examining expression levels of the two target genes, *FPGS1* and *CCoAOMT1*. Both showed less than 10% of wild-type levels in the corresponding single or double mutants (Table 2). Visualization of differentially expressed genes showed that 97 genes had the same expression patterns among mutant lines. Fifty-eight genes had changed expression only in the double mutant, 86 genes only in *ccoaomt1* and 15 genes only in *fpgs1*. The differentially expressed gene list in the double mutant substantially overlapped with that of *ccoaomt1* (Additional file 7: Fig. S4).

**Table 2:** Selected differentially expressed genes closely related to cell wall biosynthesis and C1 pathways from 6-week-old stems of *fpgs1*, *ccoaomt1*, and *fpgs1ccoaomt1* plants

AGI	Gene name/putative function	*fpgsl/WT*		*ccoaomt1/WT*		*fpgs1ccoaomt1/WT*		*fpgs1ccoaomt1/fpgs1*		*fpgs1ccoaomt1/ccoaomt1*	
		Fold change	P-value	Fold change	P-value	Fold change	P-value	Fold change	P-value	Fold change	P-value
At5g05980	*FOLYLPOLYGLUTAMATE SYNTHETASE 1 (FPGS1)*	*0.08*	0	1.12	0.1	*0.09*	0	1.10	0.7	*0.08*	0
At4g34050	*CAFFEOYL COENZYME A O-METHYLTRANSFERASE 1 (CCoA0MT1)*	1.01	0.95	*0.04*	0	*0.06*	0	*0.06*	0	1.58	0.02
Phenylpropanoid/lignin biosynthesis											
At2g44130	*KELCH REPEAT F-B0X 39 (KFB39):* phenylpropanoid metabolic process	*0.42*	1.4E−138	*0.12*	0	*0.11*	0	*0.27*	0.00	0.90	0.29
At3g59940	*KELCH REPEAT F-BOX 5O (KFB50):* phenylpropanoid metabolic process	0.64	0	*0.47*	0	*0.44*	0	0.69	1.88E−04	0.93	0.52
At1g80440	*KELCH REPEAT F-BOX 20 (KPB20):* phenylpropanoid metabolic process	1.04	0.09	*0.47*	0	0.60	0	0.58	1.17E−05	1.29	0.15
At3g53260	*PHENYLALANINE LYASE 2 (PAL2):*	1.26	4.82E−14	1.83	1.1E−14	1.73	2.3E−08	1.37	1.4E−40	0.94	0.34
At1g80820	*CINNAMOYL COA REDUCTASE (AtCCR2)*	*2.82*	0	*7.18*	0	*8.56*	0	*3.04*	4.58E−45	1.19	0.23
At1g20510	*4CL-LIKE1: OPC-8:0 COA LIGASE1 (0PCLI)*	*2.43*	0	*2.55*	0	*2.57*	0	1.06	0.60	1.01	0.93
At2g29130	*LACCASE 2 (AtLAC2)*	*0.37*	0	1.33	6.04E−07	1.26	8.61E−05	*3.45*	2.25E−102	0.95	0.83
At2g37130	*PEROXIDASE 21 (AtPrx21)*	1.27	3.94E−04	*0.44*	4.16E−33	*0.34*	0	*0.27*	2.25E−13	0.77	0.03
At4g38620	*AtMYB4:* regulation of phenylpropanoid metabolic process	0.60	0	*0.37*	0	*0.51*	0	0.86	1.26E−05	1.37	1.02E−12
Cell well synthesis											
At3g28180	*CELLULOSE-SYNTHASE LIKE C4 (AtCSLC4)*	*2.18*	0	*4.24*	0	*4.22*	0	1.93	2.81E−10	1.00	0.98
At5g57560	*CELL WALL-MODIFYING ENZYME, XYLOGLUCAN ENDOTRANSGLUCOSYLASE/HYDROLASE 22 (AtXTH22)*	*7.37*	0	*19.68*	0	*17.02*	0	*2.31*	3.24E−98	0.86	0.12
At3g16720	*RING-H2 PROTEIN, TOXICOS EN LEVADURA 2*	*3.54*	0	*3.28*	0	*5.89*	0	1.66	7.46E−22	1.79	6.18E−05
At3g45960	*EXPANSIN-LIKE A3 (EXPL3)*	*6.25*	0	*14.99*	0	*14.91*	0	*2.38*	2.90E−12	0.99	0.87
At3g45970	*EXPANSIN-LIKE A1 (EXPL1)*	*4.17*	8.27E−102	*14.41*	0	*9.51*	0	*2.28*	2.38E−07	0.66	0.03
At4g38400	*EXPANSIN-LIKE A2 (EXLA2)*	1.31	1.56E−03	*2.17*	1.41E−33	*2.37*	9.2E−46	1.82	5.93E−04	1.09	0.38
Glucosinolates metabolism											
At4g03050	*AOP3:* involved in glucosinolate biosynthesis	1.08	0.54	1.07	0.01	*0.31*	1.19E−07	*0.28*	1.27E−07	*0.29*	0
At1g21120	*COMT-LIKE GENE: INDOLE GLUCOSINOLATE METHYLTRANSFERASE 2 (IGMT2)*	1.69	0	*4.70*	0	*4.18*	0	*2.47*	0	0.89	0.15
At1g21110	*COMT-LIKE GENE: INDOLE GLUCOSINOLATE METHYLTRANSFERASE 3 (1GMT3)*	2.83	0	3.65	3.40E−127	*10.33*	0	*3.65*	0	*2.83*	0
At1g21130	*COMT-LIKE GENE: INDOLE GLUCOSINOLATE METHYLTRANSFERASE 4 (IGMT4)*	2.20	4.31E−06	1.69	0.009	*2.93*	1.32E−13	2.33	0.010	1.74	1.13E−05
One carbon metabolism											
At3g22740	*HOMOCYSTEINE S-METHYLTRANSFERASE (HMT3)*	1.59	5.29E−102	1.50	0	*0.53*	0	*033*	1.66E−29	*0.35*	0

*P*-values were obtained by associative analysis [81]. The italic highlighted genes were significant, with ratio of a 1.5 times and *P* < 2.20E−06 (a Bonferroni-corrected *P*-value cutoff)

Genes encoding proteins in the phenylpropanoid/lignin biosynthesis pathway were among the group of genes with significant expression alterations in single or double mutants. For instance, a significant number of genes encoding Kelch domain-containing F-Box (KFB) proteins, which are negative regulators mediating phenylalanine ammonia lyase (PAL) proteolytic turnover through ubiquitination-26S proteasome pathway [55, 56], changed expression in single and double mutants (Table 2). Consistent with the decreased expression of *KFB* genes, *PAL2* transcripts increased in all three mutants, although at less than the twofold ratio cutoff. In the middle and later part of the lignin biosynthesis pathway, *At1g80820* (*AtCCR2*) and *At1g20510* (*4CL*-*like1/OPC*-*8:0 CoA ligase1, OPCL1*) were upregulated in both single and double mutants. A number of *PEROXIDASE* (*Prx*) and *LACCASE* (*LAC*) genes also exhibited altered expression levels in the mutant backgrounds. For example, *At2g29130* (*AtLAC2*) was significantly downregulated in *fpgs1* and slightly upregulated in *ccoaomt1* and the double mutant. Furthermore, *At2g37130* (*AtPrx21)* was significantly downregulated in *ccoaomt1* and the double mutant while slightly upregulated in *fpgs1* (Table 2). Among the transcription factors that have been shown to be involved in the regulation of phenylpropanoid/lignin biosynthesis, expression of *AtMYB4* (*At4g38620*), a key negative regulator of the phenylpropanoid acids and esters pathway, was significantly downregulated in all three mutants. Expression levels of genes involved in other aspects of wall synthesis were also altered in the mutants. These genes included *CELLULOSE*-*SYNTHASE LIKE C4* (*AtCSLC4*), *XYLOGLUCAN ENDOTRANSGLUCOSYLASE/HYDROLASE22* (*AtXTH22*), *TOXICOS EN LEVADURA2* and three genes that encode expansin-like (EXPL) proteins (Table 2).

The number of genes differentially expressed between each single and the double mutant was very small, 10 for *fpgs1* and 21 for *ccoaomt1* (Additional file 8: Table S4). Noticeably, there was significant representation of genes involved in the downstream portions of the C1 and lignin pathways. For example, four genes that were downregulated in all three mutants were from the pathway involved in tryptophan-derived indole glucosinolate metabolism (Table 2), which is tightly linked to methionine metabolism [57]. Increase of transcripts of the glucosinolate biosynthesis genes was also seen in earlier work in Arabidopsis mutants with reduced lignin [23]. These genes included *2*-*oxoglutarate*-*dependent dioxygenase* (*AOP3*) and *indole glucosinolate methyltransferase 2*, *3*, and *4* (*IGMT2, IGMT3,* and *IGMT4*). Expression of *homocysteine S*-*methyltransferase3* (*HMT3*), which is involved in the *S*-methylmethionine (SMM) cycle responsible for the cycling of methionine and controlling the level of AdoMet in plants [58], also was reduced significantly in the double mutant compared with each of the single mutants. Other differentially expressed genes involved in phenylpropanoid/lignin biosynthesis that changed expression between the single and the double mutant included *AtCCR2*, *KFB39,* and *AtPrx21* (Table 2).

## Discussion

Previous studies have shown that single *ccoaomt1* [22, 35] and *fpgs1* [43] mutants have reduced lignin levels. Here we expanded work on analysis of these single mutants and determined whether lignin levels could be further reduced in *fpgs1ccoaomt1* double mutants without compromising plant growth. The *fpgs1ccoaomt1* double mutants had the lowest total AcBr-based lignin content and the highest sugar release when compared to WT and single-mutant plants *fpgs1* and *ccoaomt1* (Fig. 3, Table 1). The additional reduction in lignin observed in *fpgs1ccoaomt1* tissue with minimal or no impact on plant growth suggests that *FPGS1* and *CCOAOMT1* are suitable gene targets for simultaneous downregulation to further improve plants for biofuel production beyond single-gene downregulation.

To better understand how disruption of both *FPGS1* and *CCoAOMT1* led to plants with further decreased lignin content and additional sugar release, we conducted glycomics, metabolomics, and transcriptomics studies on WT, *fpgs1, ccoaomt1*, and *fpgs1ccoaomt1* stem tissue. Although data analyses revealed global differences among the various genotypes (i.e., between WT, single, and double mutants), such differences were minor. Overall, the double mutant mainly resembled the *ccoaomt1* single mutant, with minor additional changes contributed by the effects of the *FPGS1* mutation, leading to a further-improved saccharification efficiency. To allow comparison of wall analysis traits with transcriptome data using developmentally matched tissues, we completed lignin content, saccharification efficiency, and various -omic studies using green mature stem tissue. Future studies will be important to ascertain whether the double mutant maintains its improved sugar accessibility in senesced stem tissue which is most often used for biofuel production. Baxter et al. [59] noted the importance of analyzing both live green and senesced tissues for industry purposes. They found lower lignin content and greater enzymatic sugar release were consistent for both green and senesced aerial tissue of switchgrass downregulated for COMT activity.

In plants, shikimic acid from the shikimate pathway provides the backbone for synthesis of the aromatic amino acids (phenylalanine, tyrosine, and tryptophan), which serve as intermediates for a wide range of important metabolites, including lignin and folates [60, 61] (Additional file 9: Fig. S5). Phenylalanine (Phe) is the starting material for the phenylpropanoid pathway, which leads to lignin production. Significant reductions in shikimic acid and phenylalanine in all three mutants indicate that disruption of *FPGS1* and *CCoAOMT1* alone or together led to feedback adjustment at the entry point of the pathway, which could be a very efficient regulation mechanism of the phenylpropanoid pathway and its downstream branches (Additional file 9: Fig. S5). Feedback regulations of the phenylpropanoid pathway also has been observed after mutating different genes in this pathway, such as *UGT78D1/D2* or *C4H* [62–64]. Even though levels of intermediate products as well as the downstream lignin content and compositions are different between tissues from *fpgs1* and *ccoaomt1* mutant lines, the double-mutant tissue does not have additive downregulation of Phe level, indicating the feedback changes are very sensitive but with limited capacity in response to downstream changes. Although expression of most genes in the early portion of the phenylpropanoid pathway are slightly higher in the mutants, it is worth pointing out that *PAL2*, the major gene that controls the entry point to this pathway and whose expression is negatively regulated by the downstream products [62], was significantly upregulated in all three mutants. Expression of *PAL2* increased by 26%, 83%, and 73% in *fpgs1*, *ccoaomat1*, and the double mutant, respectively, compared to WT. Our results are consistent with previous observations that feedback regulation at the entry point of the phenylpropanoid pathway can occur at both transcriptional and enzyme activity levels in different plant species [62–65]. Changes in the expression of genes within the lignin biosynthetic pathway suggest a pathway-wide retuning when one or more steps of the pathway are disturbed. Often these changes were carried through a particular member of the gene family as observed by Vanholme et al. [23]. In plants, *AtCCR2* and its orthologs are considered to be involved in the biosynthesis of phenolics whose accumulation may respond readily to different conditions [66–68]. On the other hand, the main family member involved in lignin biosynthesis, *AtCCR1,* does not change expression levels even when there are changes in lignin content [23].

Differences of lignin monomer profiles in single and double mutants in comparison to WT plants indicate that regulation of different types of lignin monomer production is dynamic, as observed by studies of lignin mutants in various species [23, 69]. Modification of one step or branch of the lignin pathway will result in changes in other steps or branches of the pathway. While changes of metabolites in *fpgs1* are mostly limited to sugars, sugar alcohols, and amino acids (Additional file 5: Fig. S3), there are significant shifts in the metabolites associated with phenylpropanoid pathway in the stem from *ccoaomt1* mutant. Despite the fact that both G- and S- lignins are reduced in the *ccoaomt1* mutant stem tissue, its increased S/G ratio suggests that some S-monomer units may come from another metabolic pathway in the *ccoaomt1* plant, bypassing the CCoAOMT step. Using stable isotope labeling, sinapic acid (SA) was shown to be converted to S-lignin through sinapoyl-CoA and sinapaldehyde in *Robinia pseudoacacia* and *Nerium indicum* [70]. Although only a side pathway in the poplar and Arabidopsis [71], the path from SA to S-lignin might become more active when the main pathway to s-monomer production is impaired, as in the case of a mutation in *CCoAOMT1* gene. The increased ferulic acid (FA), SA, and sinapoyl malate levels in the *ccoaomt1* mutant provided evidence that the phenylpropanoid pathway redirects the flow of metabolites preferentially toward two downstream branches. Preferential flow toward one branch leads to the increased production of sinapoyl malate while the other branches toward S-monomer production through sinapoyl-CoA and sinapaldehyde. Similar to findings with *ccoaomt1* tissue, levels of FA, SA, and sinapoyl malate were also significantly increased in *fpgs1ccoaomt1* tissue. The lignin composition in the *fpgs1ccoaomt1* double-mutant tissue likely reflects the combined effects of each single mutant. For example, G- and S- lignin levels of *fpgs1ccoaomt1* were similar to *ccoaomt1*, while H-levels were the combined effect of each single mutant.

In addition to lignin, levels of several secondary metabolites also were altered in the single and double mutants when compared to WT. Most notable were those in the phenylpropanoic acid and ester metabolism pathways. The significant increases in ferulic acid, sinapic acid, feruloyl malate, and sinapoyl malate in both *ccoaomt1* and the double mutant are likely redirected metabolites caused by excess caffeoyl-coA that accumulated due to mutation of the *CCoAOMT1* gene. Similar increases in feruloyl malate and sinapoyl malate were observed in mutants/RNAi lines for the gene next downstream in the pathway, namely, *ccr1* mutant in Arabidopsis and *PtCCR* downregulated lines in poplar [67, 68]. Similar to poplar tissue downregulated *CCR*- and *CCoAOMT* expression [21, 68], *ccoaomt1* single and *fpgs1ccoaomt1* double mutants accumulated syringic acid-4-*O*-glucoside and vanillic acid-4-*O*-glucoside, both readily produced from the accumulated ferulic acid [67]. In these mutants, G- and S-lignin production would be interrupted and the influx of *p*-coumaroyl CoA presumably would be diverted to the biosynthesis of flavonoids such as kaempferol and quercetin as seen in the mutants in this study and early reports [23, 35] (Fig. 5). Additional modifications of metabolite content in the double mutant contributed by the *FPGS1* mutation were very few, with only two metabolites (kaempferol and guaiacyl lignan) exhibiting obvious additional increases.

The observed increase of lignan in the double mutant seemed unusual considering contradictory with the reduced lignin in all mutants. One might expect that a reduction of G-lignin would also result in a reduction in guaiacyl lignans, given that they both are derived from the same precursor [72]. However, it cannot be assumed that a reduction in G-lignin dictates or necessitates a decline in all guaiacyl lignans, because they are synthesized and are located in different tissues/organs and are likely sourced from different compartmentalized substrate pools. The G-lignin measured by thioacidolysis and NMR reported in this study is from wall-bound polymeric lignin. Guaiacyl lignan measured by GC–MS was from the ethanol-soluble metabolites pool. The overall wall-bound lignins, especially G-lignin, are one of the most abundant substrates of plant secondary cell wall. Compared to other metabolites in the GC–MS analysis, the lignan level is relatively low, ranging from 0.8 to 2 ug/g fresh weight (sorbitol equivalents), which accounts for a very small portion of coniferyl alcohol derivatives and will not likely change the overall picture of lowered G-lignin content in the mutants compared with WT nor the fact that the double mutant has less lignin than each single mutant. The reason why the guaiacyl lignan has a small but accumulated increase in the double mutants is not obviously based on the current work. We speculate that this could be attributed to the changed phenylpropanoid pathway feedback rechanneling of metabolites to other side branches. Future work focusing more deeply on ethanol-soluble lignin precursors in these mutants will be needed in order to understand the lignan regulation in relation to the wall-bound lignin modifications.

Our work provided additional insights into interactions among genes involved in lignin biosynthesis. For example, the role of the AtMYB4 transcription factor in regulating downstream lignin pathway genes is not fully understood. Although the expression of *CCoAOMT* was reduced in the *atmyb4* mutant and increased in *AtMYB4* overexpression lines [73], a direct interaction between AtMYB4 and the *CCoAOMT* promoter was not shown. It was not clear if AtMYB4 decreased the expression of a repressor of *CCoAOMT* to regulate its level or the expression of *CCoAOMT* simply responded to metabolic feedback regulation [73]. In this study, although *AtMYB4* transcript levels were reduced in all three mutants, the expression level of *CCoAOMT* was not affected in the *fpgs1* mutant tissue (Table 2). This result indicates that correlation of the expression level of *CCoAOMT* to that of *MYB4* reported earlier [73] is most likely due to metabolic feedback regulation.

The similarity in the glycome profiles among WT, *fpgs1*, *ccoaomt1*, and *fpgs1ccoaomt1* plants indicated that no major non-cellulosic glycans are modified among the single and double mutants due to *FPGS1* and *CCOAOMT1* mutations. The increased extractabilities for non-cellulosic carbohydrates from cell wall of the mutants, in particular of the double mutant, are likely attributed to lignin structure and composition changes. These changes could lead to the loosening of the cell-wall secondary structures, which enables better access to cellulose components for increased sugar release in the double mutants, especially when no pretreatment was applied. In this study, reduced lignin content among all three mutants and changed S/G ratios in both *ccoaomt1* and the double mutant indicated lignin modification is likely the main cause of reduced recalcitrance in the mutants. Changes of transcripts levels for genes encoding enzymes involved in lignin polymerization such as laccases and type III peroxidases [74–78] provide molecular evidence for lignin structure modifications. In addition, 2D HSQC-NMR analysis revealed changes in interunit linkages of the lignin in the mutants that provide direct evidence of structural alterations, derived mainly from *CCoAOMT1* mutation. Together these data provide comprehensive insights into the reduced recalcitrance and other likely changes in the mutants unrelated to lignin. With regard to the latter, global changes in the mutant uncovered by transcriptomics and glycomics reported here could guide future studies on previously undescribed physiological processes impacted by CCoAOMT1 and FPGS1.

## Conclusion

In this work, we show that additional reductions in lignin content and improved sugar release can be achieved by simultaneous downregulation of genes in the C1 (*FPGS1*) and lignin biosynthetic (*CCoAOMT1*) pathway. The double mutant exhibited a combined profile of lignin content and composition derived from each single mutant and improved sugar release. Omics data show that the double mutant resembled mostly the *ccoaomt1* mutant. Changes introduced by *FPGS1* mutation in the double mutant indicate that the lignin pathway can be further modified to achieve improved saccharification efficiency with minimal or no impact to growth in Arabidopsis. These findings can now be applied to studies with poplar, switchgrass, or other biofuel crops to determine the generality of these results across species and its applicability for the biofuel industry.

## Methods

### Plant materials and growth conditions

The *ccoaomt1* (SALK_151507) T-DNA line was obtained from the Arabidopsis Biological Resource Center (ABRC) at The Ohio State University. The *fpgs1* was the T-DNA line originally designated as *atdfb*-*1* or *fpgs1*-*1* and identified through a forward genetic screen [43, 51]. Both *ccoaomt1* and *fpgs1* lines reside in the *Columbia* (*Col*-*0*) genetic background. Double *fpgs1ccoaomt1* mutants were generated by first crossing the two single homozygous mutants. The F1 was then self-fertilized, and the resulting F2 generation genotyped at the DNA level to find the homozygous double mutants. Confirmed double homozygous plants were self-fertilized to obtain seed used for the experiments. Wild-type and homozygous *fpgs1*, *ccoaomt1,* and *fpgs1ccoaomt1* plants were confirmed by RT-PCR where the mutant lacks amplifications for its relevant target transcripts. The primers used for genotyping and RT-PCR are listed in Additional file 1: Table S1. All plants analyzed were grown in the same growth chamber to ensure uniform environmental conditions (16/8 h light/dark, 100 µmol photons m^−2^ s^−1^ light, 60% relative humidity, and temperature of 23 °C in the day, 21 °C at night) [43, 75].

### Phenotypic characterization of *fpgs1ccoaomt1* double mutant and preparation of plant material for analysis

Seeds of all genotypes were vertically grown on 0.7% agar plates supplemented with half strength Murashige and Skoog medium (pH 5.7) containing 1% (w/v) sucrose for roots phenotyping [51]. Another set of seeds was directly sown into soil and grown in the growth chamber for plant tissue collections [79]. Arabidopsis plants were grown in a mixture of Metro-Mix 350 (Sun Gro Horticulture, Agawam, Massachusetts) and Metro-Mix 830 (Sun Gro Horticulture, Agawam, Massachusetts) at a 1-to-1 ratio. The pH of the mixture was in the 5.4–7.5 range. Fertilizer was not added at the early stage because all Metro-Mix formulas contain a starter nutrient level sufficient for Arabidopsis seedling development. When the plants started to bolt, they were watered with solution supplemented with fertilizer (Peters 20–10–20 = N–P–K) as needed [80]. When the plants were 6 weeks old and the first silique started to turn yellow, the aboveground part of the plants was collected to quantify whole plant and stem fresh weight. The stems, which were devoid of leaves, siliques, and flowers, were dried for lignin, sugar release, and glycome-profiling analyses. The stem materials for microarray and metabolite analysis were collected directly from the plants in the growth chamber by removing the leaves, siliques, and flowers, and then immediately frozen in liquid nitrogen. The plant material used for each analysis had at least three biological replicates with each replicate pooled from at least 20 plants.

### RT-PCR, qRT-PCR, and microarray analysis

Nine-day-old plate-grown wild-type, *fpgs1*, *ccoaomt1,* and *fpgs1ccoaomt1* whole seedlings were collected for RT-PCR analysis to analyze target genes *FPGS1* and *CCoAOMT1* expression. Wild-type Arabidopsis plants at two stages of development were used for gene expression analysis by real-time qRT-PCR. Whole seedlings of 2-week-old plate-grown Arabidopsis plants and 6-week-old mature Arabidopsis plant tissue were used for RNA isolation. For the latter, stem tissue from various portions of the plant were sampled as shown in Fig. 1. For microarray analysis, whole stem tissue without leaves and flower parts were harvested from 6-week-old wild-type, *fpgs1*, *ccoaomt1,* and *fpgs1ccoaomt1* plants.

Total RNA was isolated by Spectrum Plant Total RNA Kit (Sigma-Aldrich) and Turbo DNase treatment was applied to remove DNA contamination. The cDNA was synthesized by Superscript III Reverse Transcriptase (Invitrogen). qRT-PCR was run on a 7900HT, and the data were analyzed by SDS 2.4.1 software (machine and software, Applied Biosystems, Foster City, California).

Total RNA from 6-week-old mature stems of WT, single, and double mutants was used for microarray analysis. RNA quantification and quality control were conducted by Bioanalyzer, NanoDrop, and Qubit. Affymetrix Arabidopsis ATH1 Genome Array was used for labeling. The experiment was performed according to manufacturer’s instructions. Microarray data were submitted to ArrayExpress with accession number of E-MTAB-7536. Data were normalized using Robust Multichip Average algorithms. Differentially expressed (DE) genes were selected using associative analysis [81] from *fpgs1*, *ccoaomt1,* and *fpgs1ccoaomt1* using a ratio cutoff of two and P-value cutoff of 2.19 × 10^−6^ (derived from Bonferroni-corrected *P* value (0.05/22,810) of the ATH1 chip). The selected genes were first preprocessed to one of the Cytoscape-supported XML-based exchange formats by using a custom PYTHON script. This XML file was imported to Cytoscape [82] and visualized by using yFiles Layouts. All up- and downregulated genes were highlighted in red and blue, respectively.

### Determination of lignin composition and acetyl bromide (AcBr) lignin content

Stems harvested from 6-week-old mature plants were dried in a 37 °C oven for 7 days and then ground by 20 Mesh Wiley mill. The ground materials were extracted through successive steps by methanol, chloroform: methanol (2:1 v/v), methanol, and water to acquire alcohol-insoluble residue (AIR) [30]. Around 20 mg AIR was used to determine lignin content by the acetyl bromide (AcBr) method [52, 83]. The thioacidolysis method was used to determine lignin composition. Approximately 15 mg AIR was used to produce lignin monomers by thioacidolysis. The lignin-derived monomers (S, G, H) were identified and quantified by gas chromatography mass spectrometry (GC/MS) as described previously [84, 85]. The relative content for each lignin monomer obtained by thioacidolysis analysis was calculated for every 100 mg of AIR.

### Analysis of total sugar and enzymatic sugar

The AIR material for lignin analysis was also used for total sugar and enzymatic sugar determination according to previously described procedures [30, 43]. To focus on total hydrates from lignocellulose and not total cell polysaccharides, the AIR material was destarched using amylase assay solution (α-amylase [Sigma): amyloglucosidase [Sigma] = 1:1 in citrate buffer [0.1 M, pH 5.0]). To release total sugar from all wall carbohydrates, about 7.5 mg destarched AIR material was treated with 72% H_2_SO_4_ at 30 °C for 1 h, then diluted by water, prior being autoclaved at 121 °C for 1 h to obtain simple sugars. Sugar content was analyzed using the phenol–sulfuric acid sugar assay [30, 86].

For enzymatic hydrolysis sugar release analysis, material was digested with a cellulase enzyme mix to ensure sugars from cellulose were released, as described previously [43]: to summarize, approximately 10 mg destarched AIR material was digested in 20 ul of a cellulase stock from *Trichoderma reesei* (Sigma-Aldrich) and Novozyme 188 stock from *Aspergillus niger* (Sigma-Aldrich) mixture in a 1:1 ratio in 2.5 ml citrate buffer (0.1 M, pH 5.0) at 50 °C for 72 h. Enzymatic hydrolyzed sugar content was measured by the phenol–sulfuric acid sugar assay [30, 86]. This analysis method was described in detail in our previous publication [43].

### Whole cell-wall NMR analysis and glycome profiling

Using nuclear magnetic resonance (NMR) technology [87, 88], the degree of the complex structures of lignin polymers was examined for the mutants and wild-type plants. The AIR material for lignin analysis was also used for whole cell-wall NMR analysis and glycome profiling.

For whole cell-wall NMR analysis, the AIR material was extracted by ethanol/toluene solvent (1:2 v/v) for 12 h and vacuum-dried for 48 h. The extract-free samples were ball-milled at 580 rpm for 4 h. About 80 mg of ball-milled biomass was dissolved/soaked in 0.5 mL of DMSO-d6/HMPA-d18 for NMR analysis. NMR spectra were acquired at 298 K using a Bruker Avance III 400 MHz console equipped with a 5-mm BBO probe. The central DMSO solvent peaks (δH/δC = 2.49/39.5 ppm) were used for chemical shift calibration. Two-dimensional 1H-13C heteronuclear single-quantum coherence (HSQC) spectra were collected using a Bruker standard pulse sequence (‘hequetgpsi2’). Volume integration of cross peaks in HSQC spectra was carried out using Bruker’s TopSpin 3.5 software.

For glycome profiling, the AIR material was extracted sequentially with increasingly harsh reagents [89]. The sequential resultant extracts were analyzed for carbohydrates by phenol–sulphuric acid assays and were also screened with a large collection of plant glycan-directed monoclonal antibodies by enzyme-linked immunosorbent assays, as described previously [89]. Briefly, each extraction step consists of increasingly harsh reagents in the following order: ammonium oxalate (AO, 1st), sodium carbonate (2nd), 1 M KOH (3rd), 4 M KOH (4th), acidic sodium chlorite (5th), and 4 M KOH PC (postchlorite treatment, 6th) [89–91]. Presence of each carbohydrate epitope was detected by the enzyme-linked immunosorbent assay (ELISA) using glycan-directed monoclonal antibodies (mAbs) against respective cell-wall extracts.

### Metabolite profiling

Approximately 90 mg of ground 6-week-old fresh stem material was weighed into a 15-ml centrifuge tube containing 2 ml of 80% ethanol and 15 ul of sorbitol (1 mg/ml) added as an internal standard. Samples were extracted at RT overnight with end-over-end rotation, centrifuged at 4500 rpm for 20 min, and then decanted into scintillation vials that were stored at − 20 °C. An additional 1 ml of 80% ethanol was added to the plant residue and a second extraction was performed at RT overnight. After centrifugation, the second extract was combined with the first extract and mixed well, and 2 ml of the combined extract was dried under nitrogen. The dried extract was dissolved in 0.5 ml acetonitrile and silylated to generate trimethylsilyl derivatives, as described previously [92]. After 2 days, 1ul aliquots were injected into an Agilent 5975C inert XL gas chromatograph-mass spectrometer (GC–MS). The single quadrupole GC–MS was operated in the electron impact (70 eV) mode, targeting 2.5 full-spectrum (50–650 Da) scans per second, as described previously [92]. Metabolomic measurements were made, as described previously [92].

## Additional files

**Additional file 1: Table S1.** Primers used for genotyping and gene expression analysis by RT-PCR and QRT-PCR.

**Additional file 2: Fig. S1.** The phenotype of 9-day-old seedlings of WT, *fpgs1*, *ccoaomt1* and *fpgs1ccoaomt1* plants. Similar to *fpgs1* single mutants, *fpgs1ccoaomt1*double mutants had short roots during early development.

**Additional file 3: Table S2.** Whole cell wall NMR analysis of 6-week-old stems of WT*, fpgs1*, *ccoaomt1* and *fpgs1ccoaomt1* plants. Note. Linkages = (β-*O*-4) + (β–β) + (β-5) *: significantly different than wild type according to one-way ANOVA analysis (*P* ≤ 0.05). **Both acetyl and methoxyl content were estimated from whole cell wall components in the AIR. To obtain changes in content between genotypes in relation to lignin, values in each sample were normalized against their own lignin using “acetyl (or methoxyl) peak area/total lignin subunits (S + G+H)”. The *f1*-*1*, *f1*-*2* and *f1*-*3* are three biological replicates for *fpgs1*; the *cc1*-*1*, *2, 3* are three biological replicates for *ccoaomt1*; the *f1cc1*-*1, 2, 3* are three biological replicates for *fpgs1ccoaomt1.*

**Additional file 4: Fig S2.** Heatmap of glycome profiling analysis of stem extracts from 6-week-old stem of WT, *fpgs1*, *ccoaomt1* and *fpgs1ccoaomt1* plants. AIR was processed through six different extraction conditions with increasing harshness. Only epitopes of significantly different antibody detection signals in pairwise comparisons among these four genotypes were included in this heatmap (Student’s *t*-test of *P*-value ≤ 0.05). Color represent the signal level of different monoclonal antibodies (mAbs) bound with non-cellulosic cell wall epitopes. The name of the antibodies and their recognized non-cellulosic carbohydrate epitope types are presented on the right side of the heat map.

**Additional file 5: Fig. S3.** The heatmap of log2 ratio of metabolites from 6-week-old stem of *fpgs1*, *ccoaomt1* and *fpgs1ccoaomt1* compared with WT. More than 100 peaks were detected in WT*, fpgs1*, *ccoaomt1* and *fpgs1ccoaomt1,* and 77 were assigned to known chemical structures. The identified compounds were arranged based on their categories, and their relative level changes in *fpgs1*, *ccoaomt1* and *fpgs1ccoaomt1* compared to WT were visualized as a heatmap by software MeV. Compound 2-*O*-feruloyl-malic acid was not detected in WT and *fpgs1*, so it is not shown on the heatmap. Abbreviations: *f1*- *fpgs1*, *cc1*- *ccoaomt1*, *f1cc1* - *fpgs1ccoaomt1*. Number positioned in front of tentatively identified metabolites are the retention time (RT; min) and key mass-to-charge (m/z) ratios.

**Additional file 6: Table S3.** Differentially-accumulated metabolites in 6-week-old stems of WT, *fpgs1 (f1)*, *ccoaomt1 (cc1)* and *fpgs1ccoaomt1 (f1cc1)* plants. Selection threshold for significant metabolites (marked with *, up in red, down in blue) is *P* < 0.05 (Student’s t-test). Metabolite concentration (ug/g fresh weight; sorbitol equivalents). The retention time (RT; min) and key mass-to-charge (m/z) ratios are positioned in front of tentatively identified metabolites.

**Additional file 7: Fig. S4.** Presentation of differentially expressed genes in the 6-week-old Arabidopsis stems of *fpgs1*, *ccoaomt1* and *fpgs1ccoaomt1* plants compared with WT. Genes with two fold changes in expression levels in *fpgs1*, *ccoaomt1* or *fpgs1ccoaomt1*, compared with WT, were included in the image. Red - upregulated genes; Blue - downregulated genes. Yellow - each mutants, square - genes in phenylpropanoid/lignin/glucosinolate pathway (At1g80820-*AtCCR2*; At1g20510-*OPCL1*; At2g29130-*AtLAC2*; At1g21110-*IGMT3*; At1g21120-*IGMT2*; At4g34050-*CCoAOMT1*; At1g21130-*IGMT4*); Triangle - one carbon pathway genes (At5g05980-*FPGS1*). Lines show the relationship of genes among the mutants.

**Additional file 8: Table S4.** Microarray analysis representing 6-week-old stems of WT, *fpgs1*, *ccoaomt1* and *fpgs1ccoaomt1* plants.

**Additional file 9: Fig. S5.** Schematic representation of shikimate pathway, aromatic amino acids (AAA) pathway, and lignin pathway association with C1 metabolism. Adapted from the following papers [13, 17, 37, 43, 93–98]. Solid Arrows represent enzymatic reactions together with the name of the enzyme that catalyzes the associated reaction. Empty circles represent the products of the enzymatic reactions along with the products’ names nearby. Dotted arrows represent multiple enzymatic reactions. Red dotted rectangles represent shikimate pathways. Amber dotted rectangles represent aromatic amino acids (AAA) pathways. Green dotted rectangles represent lignin pathways. Purple dotted rectangles represent C1 metabolism. CM: chorismatemutase; PAT: prephenate aminotransferase; ADT: arogenatedehydratase; PAL: phenylalanine ammonia-lyase; C4H: cinnamate-4-hydroxylase; 4CL: 4-coumarateCoAligase; CCR: cinnamoyl-CoA reductase; CAD: cinnamyl alcohol dehydrogenase; C3H: *p*-Coumarate 3-hydroxylase; HCT: folylpolyglutamate synthase; C3′H: *p-*coumaroyl quinate/shikimate 3′-hydroxylase, CSE: 5,10-methylene tetrahydrofolate polyglutamates; COMT: caffeic acid O-methyl transferase; CCoAOMT: caffeoyl-CoA O-methyltransferase; F5H: ferulate 5-hydroxylase; CAD: cinnamyl alcohol dehydrogenase; SGT: sinapate:UDP-glucose glucosyltransferase; SMT: sinapoylglucose:malate sinapoyltransferase; ADC: amino deoxychorismate; ADCL: amino deoxychorismate (ADC) lyase; ADCS: amino deoxychorismate (ADC) synthase; pABA: para-aminobenzoic acid; DHPS: dihydropteroate (DHP) synthase; DHFS: dihydrofolate (DHF) synthase; DHF-Glu1: dihydrofolate with one glutamate; DHFR: dihydrofolate (DHF) reductase; THF-Glu1: tetrahydrofolate with one glutamate; FPGS: folylpolyglutamate synthase; THF-Glun: tetrahydrofolate polyglutamates; SHMT: serine hydroxymethyl transferase; CH2-THF-Glun: 5,10-methylene tetrahydrofolate polyglutamates; DHC: 5,10-methylene tetrahydrofolate dehydrogenase/5,10-methenyl THF cyclohydrolase; 10-CHO-THF-Glun: 10-methenyl tetrahydrofolate polyglutamates; FTHS: 10-formyltetrahydrofolate synthetase; MTHFR: methylene tetrahydrofolate reductase; CH3-THF-Glun: 5-methyl tetrahydrofolate polyglutamates; MS: methionine synthase; SAMS: S-adenosylmethionine synthetase; SAM: S-Adenosyl methionine (AdoMet); SAMMT: S-adenosylmethionine methyltransferase; SAHH: S-adenosylhomocysteine hydrolase.
