# Supplementary material for: Combining loss of function of FOLYLPOLYGLUTAMATE SYNTHETASE1 and CAFFEOYL-COA 3-O-METHYLTRANSFERASE1 for lignin reduction and improved saccharification efficiency in Arabidopsis thaliana
Source: Biotechnol Biofuels. 2019 May 3;12:108. doi: 10.1186/s13068-019-1446-3 (PMC6498598; doi:10.1186/s13068-019-1446-3)
Supplement: Supplementary file 1 — Additional file 1: Table S1. Primers used for genotyping and gene expression analysis by RT-PCR and QRT-PCR. [file 13068_2019_1446_MOESM1_ESM.pptx]

## Slide 1
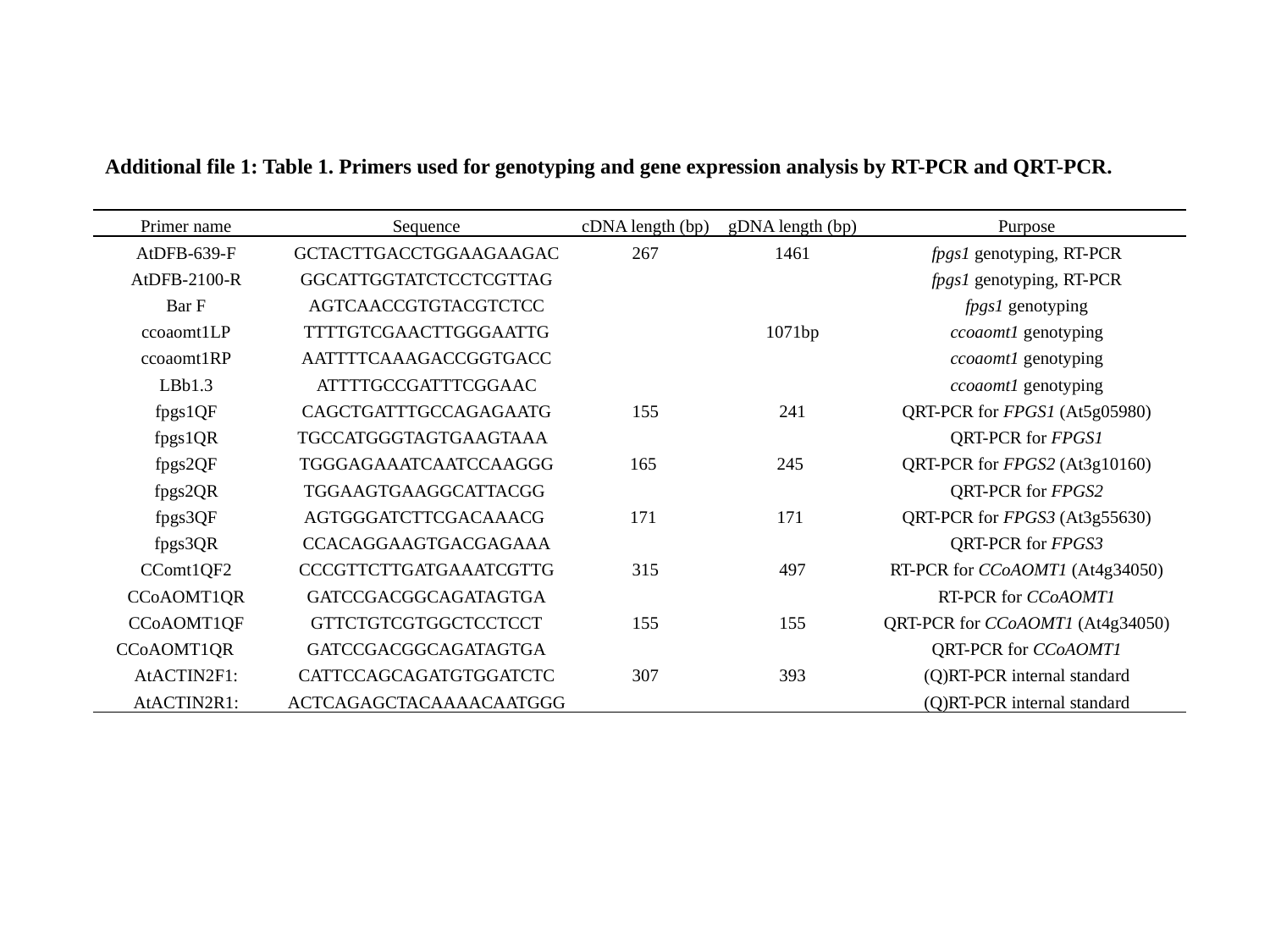

Additional file 1: Table 1. Primers used for genotyping and gene expression analysis by RT-PCR and QRT-PCR.
| Primer name | Sequence | cDNA length (bp) | gDNA length (bp) | Purpose |
| --- | --- | --- | --- | --- |
| AtDFB-639-F | GCTACTTGACCTGGAAGAAGAC | 267 | 1461 | fpgs1 genotyping, RT-PCR |
| AtDFB-2100-R | GGCATTGGTATCTCCTCGTTAG | | | fpgs1 genotyping, RT-PCR |
| Bar F | AGTCAACCGTGTACGTCTCC | | | fpgs1 genotyping |
| ccoaomt1LP | TTTTGTCGAACTTGGGAATTG | | 1071bp | ccoaomt1 genotyping |
| ccoaomt1RP | AATTTTCAAAGACCGGTGACC | | | ccoaomt1 genotyping |
| LBb1.3 | ATTTTGCCGATTTCGGAAC | | | ccoaomt1 genotyping |
| fpgs1QF | CAGCTGATTTGCCAGAGAATG | 155 | 241 | QRT-PCR for FPGS1 (At5g05980) |
| fpgs1QR | TGCCATGGGTAGTGAAGTAAA | | | QRT-PCR for FPGS1 |
| fpgs2QF | TGGGAGAAATCAATCCAAGGG | 165 | 245 | QRT-PCR for FPGS2 (At3g10160) |
| fpgs2QR | TGGAAGTGAAGGCATTACGG | | | QRT-PCR for FPGS2 |
| fpgs3QF | AGTGGGATCTTCGACAAACG | 171 | 171 | QRT-PCR for FPGS3 (At3g55630) |
| fpgs3QR | CCACAGGAAGTGACGAGAAA | | | QRT-PCR for FPGS3 |
| CComt1QF2 | CCCGTTCTTGATGAAATCGTTG | 315 | 497 | RT-PCR for CCoAOMT1 (At4g34050) |
| CCoAOMT1QR | GATCCGACGGCAGATAGTGA | | | RT-PCR for CCoAOMT1 |
| CCoAOMT1QF | GTTCTGTCGTGGCTCCTCCT | 155 | 155 | QRT-PCR for CCoAOMT1 (At4g34050) |
| CCoAOMT1QR | GATCCGACGGCAGATAGTGA | | | QRT-PCR for CCoAOMT1 |
| AtACTIN2F1: | CATTCCAGCAGATGTGGATCTC | 307 | 393 | (Q)RT-PCR internal standard |
| AtACTIN2R1: | ACTCAGAGCTACAAAACAATGGG | | | (Q)RT-PCR internal standard |
