# Supplementary material for: Combining loss of function of FOLYLPOLYGLUTAMATE SYNTHETASE1 and CAFFEOYL-COA 3-O-METHYLTRANSFERASE1 for lignin reduction and improved saccharification efficiency in Arabidopsis thaliana
Source: Biotechnol Biofuels. 2019 May 3;12:108. doi: 10.1186/s13068-019-1446-3 (PMC6498598; doi:10.1186/s13068-019-1446-3)
Supplement: Supplementary file 2 — Additional file 2: Fig. S1. The phenotype of 9-day-old seedlings of WT, fpgs1, ccoaomt1 and fpgs1ccoaomt1 plants. Similar to fpgs1 single mutants, fpgs1ccoaomt1double mutants had short roots during early development. [file 13068_2019_1446_MOESM2_ESM.pptx]

## Slide 1
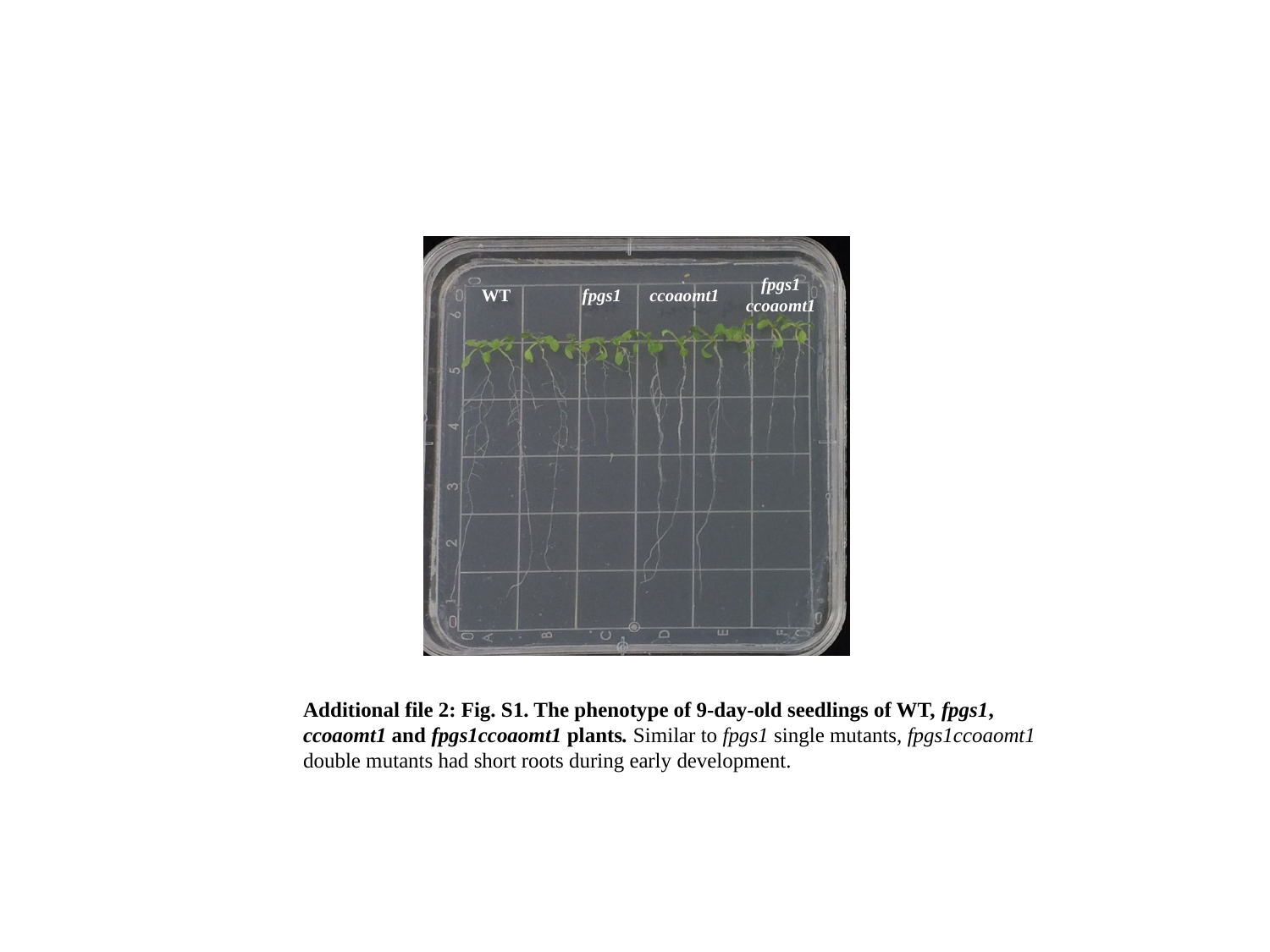

fpgs1
ccoaomt1
ccoaomt1
fpgs1
WT
Additional file 2: Fig. S1. The phenotype of 9-day-old seedlings of WT, fpgs1, ccoaomt1 and fpgs1ccoaomt1 plants. Similar to fpgs1 single mutants, fpgs1ccoaomt1 double mutants had short roots during early development.
