# Supplementary material for: Combining loss of function of FOLYLPOLYGLUTAMATE SYNTHETASE1 and CAFFEOYL-COA 3-O-METHYLTRANSFERASE1 for lignin reduction and improved saccharification efficiency in Arabidopsis thaliana
Source: Biotechnol Biofuels. 2019 May 3;12:108. doi: 10.1186/s13068-019-1446-3 (PMC6498598; doi:10.1186/s13068-019-1446-3)
Supplement: Supplementary file 3 — Additional file 3: Table S2. Whole cell wall NMR analysis of 6-week-old stems of WT, fpgs1, ccoaomt1 and fpgs1ccoaomt1 plants. Note. Linkages = (β-O-4) + (β–β) + (β-5) *: significantly different than wild type according to one-way ANOVA analysis (P ≤ 0.05). **Both acetyl and methoxyl content were estimated from whole cell wall components in the AIR. To obtain changes in content between genotypes in relation to lignin, values in each sample were normalized against their own lignin using “acetyl (or methoxyl) peak area/total lignin subunits (S + G+H)”. The f1-1, f1-2 and f1-3 are three biological replicates for fpgs1; the cc1-1, 2, 3 are three biological replicates for ccoaomt1; the f1cc1-1, 2, 3 are three biological replicates for fpgs1ccoaomt1. [file 13068_2019_1446_MOESM3_ESM.pptx]

## Slide 1
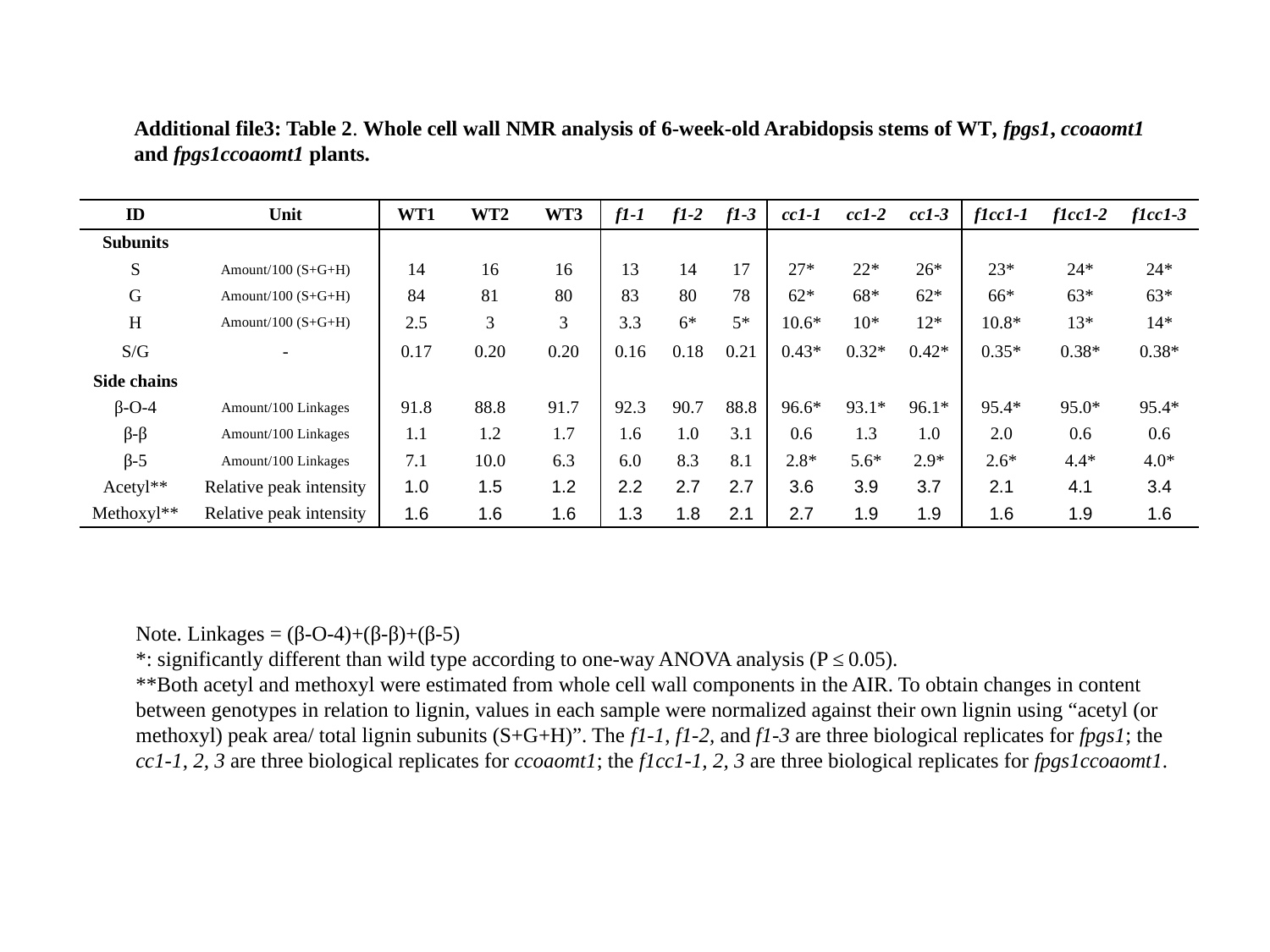

Additional file3: Table 2. Whole cell wall NMR analysis of 6-week-old Arabidopsis stems of WT, fpgs1, ccoaomt1 and fpgs1ccoaomt1 plants.
| ID | Unit | WT1 | WT2 | WT3 | f1-1 | f1-2 | f1-3 | cc1-1 | cc1-2 | cc1-3 | f1cc1-1 | f1cc1-2 | f1cc1-3 |
| --- | --- | --- | --- | --- | --- | --- | --- | --- | --- | --- | --- | --- | --- |
| Subunits | | | | | | | | | | | | | |
| S | Amount/100 (S+G+H) | 14 | 16 | 16 | 13 | 14 | 17 | 27\* | 22\* | 26\* | 23\* | 24\* | 24\* |
| G | Amount/100 (S+G+H) | 84 | 81 | 80 | 83 | 80 | 78 | 62\* | 68\* | 62\* | 66\* | 63\* | 63\* |
| H | Amount/100 (S+G+H) | 2.5 | 3 | 3 | 3.3 | 6\* | 5\* | 10.6\* | 10\* | 12\* | 10.8\* | 13\* | 14\* |
| S/G | - | 0.17 | 0.20 | 0.20 | 0.16 | 0.18 | 0.21 | 0.43\* | 0.32\* | 0.42\* | 0.35\* | 0.38\* | 0.38\* |
| Side chains | | | | | | | | | | | | | |
| β-O-4 | Amount/100 Linkages | 91.8 | 88.8 | 91.7 | 92.3 | 90.7 | 88.8 | 96.6\* | 93.1\* | 96.1\* | 95.4\* | 95.0\* | 95.4\* |
| β-β | Amount/100 Linkages | 1.1 | 1.2 | 1.7 | 1.6 | 1.0 | 3.1 | 0.6 | 1.3 | 1.0 | 2.0 | 0.6 | 0.6 |
| β-5 | Amount/100 Linkages | 7.1 | 10.0 | 6.3 | 6.0 | 8.3 | 8.1 | 2.8\* | 5.6\* | 2.9\* | 2.6\* | 4.4\* | 4.0\* |
| Acetyl\*\* | Relative peak intensity | 1.0 | 1.5 | 1.2 | 2.2 | 2.7 | 2.7 | 3.6 | 3.9 | 3.7 | 2.1 | 4.1 | 3.4 |
| Methoxyl\*\* | Relative peak intensity | 1.6 | 1.6 | 1.6 | 1.3 | 1.8 | 2.1 | 2.7 | 1.9 | 1.9 | 1.6 | 1.9 | 1.6 |
Note. Linkages = (β-O-4)+(β-β)+(β-5)
*: significantly different than wild type according to one-way ANOVA analysis (P ≤ 0.05).
**Both acetyl and methoxyl were estimated from whole cell wall components in the AIR. To obtain changes in content between genotypes in relation to lignin, values in each sample were normalized against their own lignin using “acetyl (or methoxyl) peak area/ total lignin subunits (S+G+H)”. The f1-1, f1-2, and f1-3 are three biological replicates for fpgs1; the cc1-1, 2, 3 are three biological replicates for ccoaomt1; the f1cc1-1, 2, 3 are three biological replicates for fpgs1ccoaomt1.
