# Supplementary material for: Combining loss of function of FOLYLPOLYGLUTAMATE SYNTHETASE1 and CAFFEOYL-COA 3-O-METHYLTRANSFERASE1 for lignin reduction and improved saccharification efficiency in Arabidopsis thaliana
Source: Biotechnol Biofuels. 2019 May 3;12:108. doi: 10.1186/s13068-019-1446-3 (PMC6498598; doi:10.1186/s13068-019-1446-3)
Supplement: Supplementary file 4 — Additional file 4: Fig S2. Heatmap of glycome profiling analysis of stem extracts from 6-week-old stem of WT, fpgs1, ccoaomt1 and fpgs1ccoaomt1 plants. AIR was processed through six different extraction conditions with increasing harshness. Only epitopes of significantly different antibody detection signals in pairwise comparisons among these four genotypes were included in this heatmap (Student’s t-test of P-value ≤ 0.05). Color represent the signal level of different monoclonal antibodies (mAbs) bound with non-cellulosic cell wall epitopes. The name of the antibodies and their recognized non-cellulosic carbohydrate epitope types are presented on the right side of the heat map. [file 13068_2019_1446_MOESM4_ESM.pptx]

## Slide 1
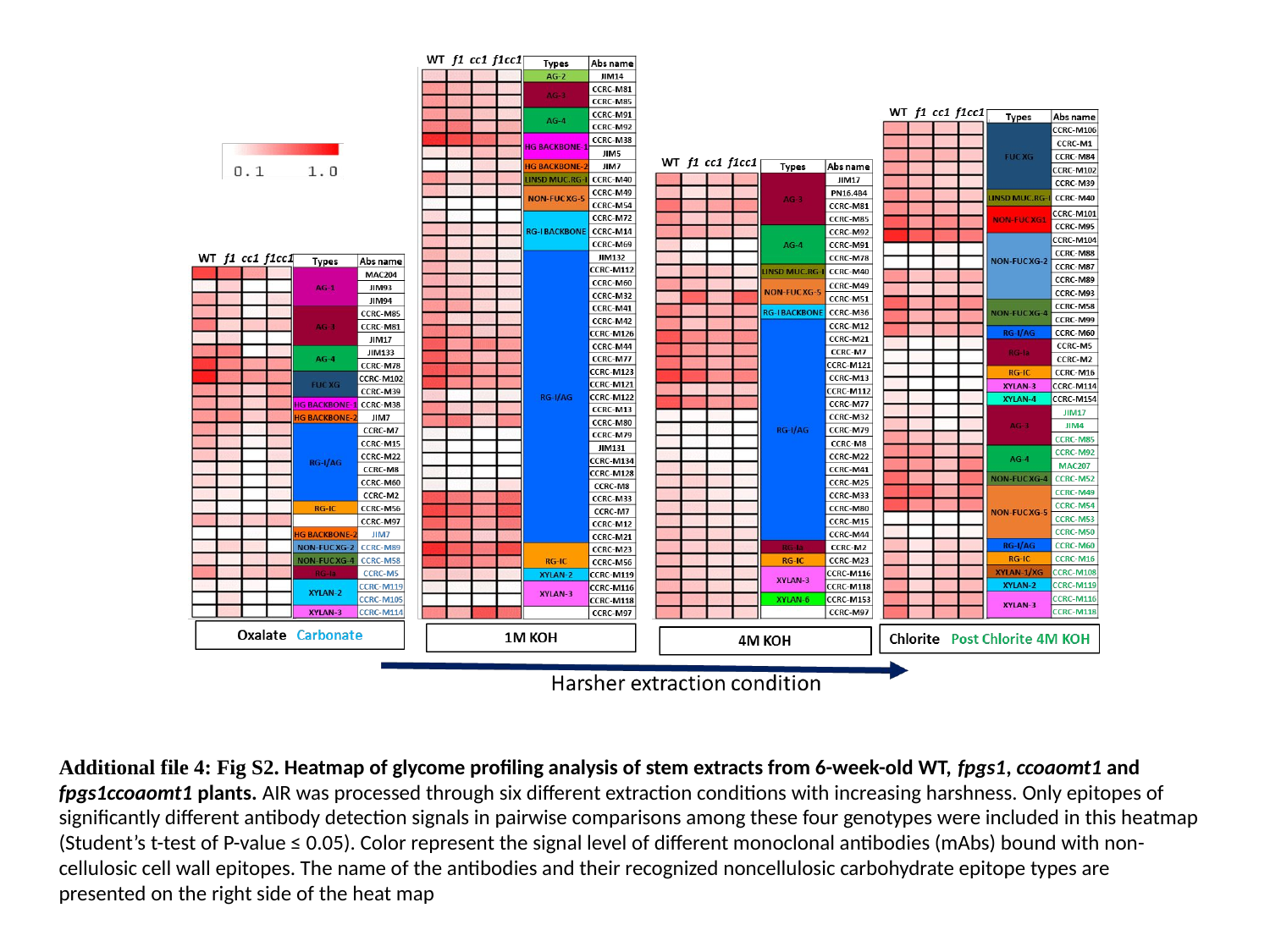

Additional file 4: Fig S2. Heatmap of glycome profiling analysis of stem extracts from 6-week-old WT, fpgs1, ccoaomt1 and fpgs1ccoaomt1 plants. AIR was processed through six different extraction conditions with increasing harshness. Only epitopes of significantly different antibody detection signals in pairwise comparisons among these four genotypes were included in this heatmap (Student’s t-test of P-value ≤ 0.05). Color represent the signal level of different monoclonal antibodies (mAbs) bound with non-cellulosic cell wall epitopes. The name of the antibodies and their recognized noncellulosic carbohydrate epitope types are presented on the right side of the heat map
