# Supplementary material for: Combining loss of function of FOLYLPOLYGLUTAMATE SYNTHETASE1 and CAFFEOYL-COA 3-O-METHYLTRANSFERASE1 for lignin reduction and improved saccharification efficiency in Arabidopsis thaliana
Source: Biotechnol Biofuels. 2019 May 3;12:108. doi: 10.1186/s13068-019-1446-3 (PMC6498598; doi:10.1186/s13068-019-1446-3)
Supplement: Supplementary file 5 — Additional file 5: Fig. S3. The heatmap of log2 ratio of metabolites from 6-week-old stem of fpgs1, ccoaomt1 and fpgs1ccoaomt1 compared with WT. More than 100 peaks were detected in WT, fpgs1, ccoaomt1 and fpgs1ccoaomt1, and 77 were assigned to known chemical structures. The identified compounds were arranged based on their categories, and their relative level changes in fpgs1, ccoaomt1 and fpgs1ccoaomt1 compared to WT were visualized as a heatmap by software MeV. Compound 2-O-feruloyl-malic acid was not detected in WT and fpgs1, so it is not shown on the heatmap. Abbreviations: f1- fpgs1, cc1- ccoaomt1, f1cc1 - fpgs1ccoaomt1. Number positioned in front of tentatively identified metabolites are the retention time (RT; min) and key mass-to-charge (m/z) ratios. [file 13068_2019_1446_MOESM5_ESM.pptx]

## Slide 1
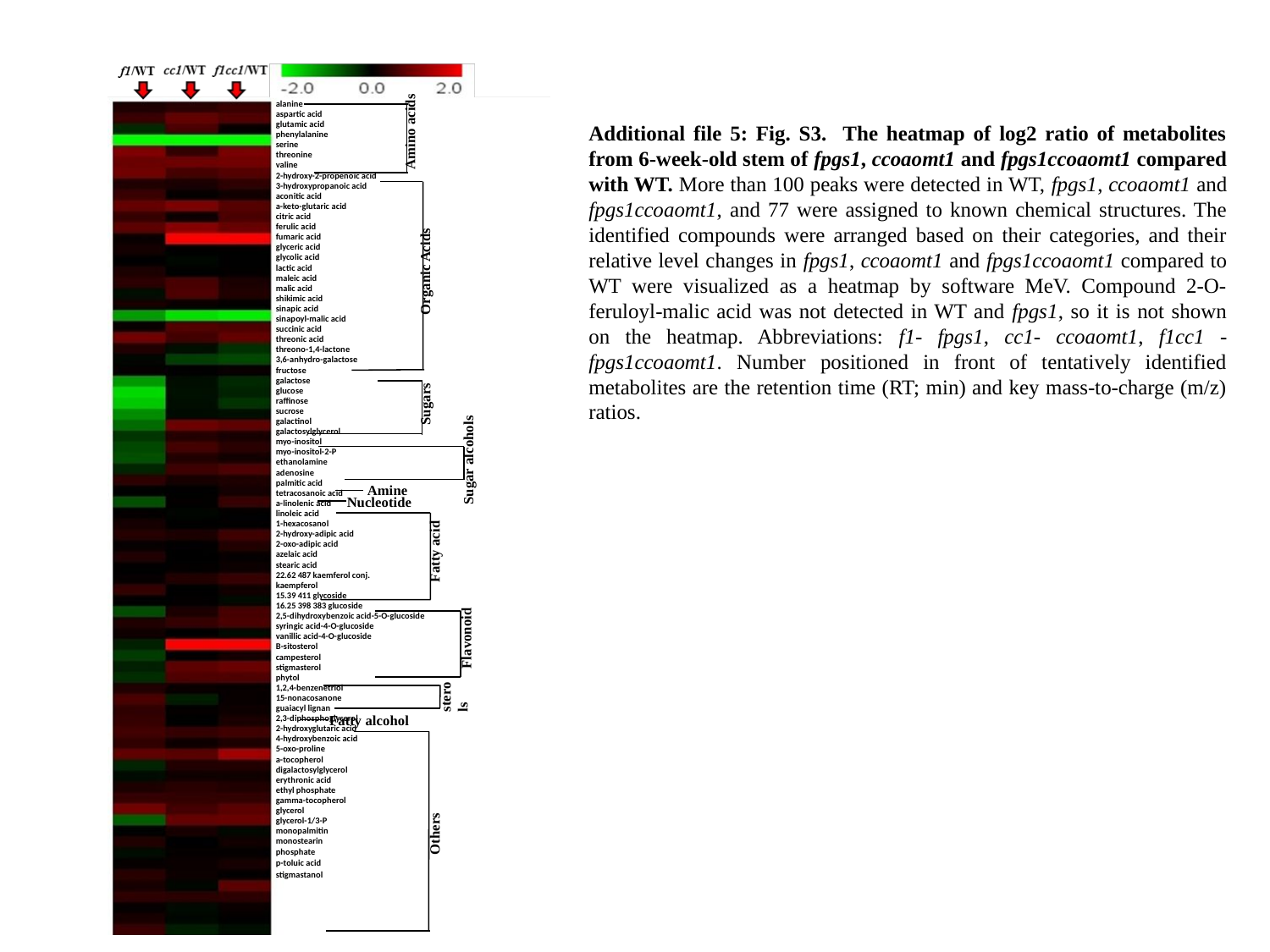

Amino acids
Organic Acids
Sugars
Sugar alcohols
Amine
Nucleotide
Fatty acid
Flavonoid
sterols
Others
Fatty alcohol
| alanine |
| --- |
| aspartic acid |
| glutamic acid |
| phenylalanine |
| serine |
| threonine |
| valine |
| 2-hydroxy-2-propenoic acid |
| 3-hydroxypropanoic acid |
| aconitic acid |
| a-keto-glutaric acid |
| citric acid |
| ferulic acid |
| fumaric acid |
| glyceric acid |
| glycolic acid |
| lactic acid |
| maleic acid |
| malic acid |
| shikimic acid |
| sinapic acid |
| sinapoyl-malic acid |
| succinic acid |
| threonic acid |
| threono-1,4-lactone |
| 3,6-anhydro-galactose |
| fructose |
| galactose |
| glucose |
| raffinose |
| sucrose |
| galactinol |
| galactosylglycerol |
| myo-inositol |
| myo-inositol-2-P |
| ethanolamine |
| adenosine |
| palmitic acid |
| tetracosanoic acid |
| a-linolenic acid |
| linoleic acid |
| 1-hexacosanol |
| 2-hydroxy-adipic acid |
| 2-oxo-adipic acid |
| azelaic acid |
| stearic acid |
| 22.62 487 kaemferol conj. |
| kaempferol |
| 15.39 411 glycoside |
| 16.25 398 383 glucoside |
| 2,5-dihydroxybenzoic acid-5-O-glucoside |
| syringic acid-4-O-glucoside |
| vanillic acid-4-O-glucoside |
| B-sitosterol |
| campesterol |
| stigmasterol |
| phytol |
| 1,2,4-benzenetriol |
| 15-nonacosanone |
| guaiacyl lignan |
| 2,3-diphosphoglycerol |
| 2-hydroxyglutaric acid |
| 4-hydroxybenzoic acid |
| 5-oxo-proline |
| a-tocopherol |
| digalactosylglycerol |
| erythronic acid |
| ethyl phosphate |
| gamma-tocopherol |
| glycerol |
| glycerol-1/3-P |
| monopalmitin |
| monostearin |
| phosphate |
| p-toluic acid |
| stigmastanol |
Additional file 5: Fig. S3. The heatmap of log2 ratio of metabolites from 6-week-old stem of fpgs1, ccoaomt1 and fpgs1ccoaomt1 compared with WT. More than 100 peaks were detected in WT, fpgs1, ccoaomt1 and fpgs1ccoaomt1, and 77 were assigned to known chemical structures. The identified compounds were arranged based on their categories, and their relative level changes in fpgs1, ccoaomt1 and fpgs1ccoaomt1 compared to WT were visualized as a heatmap by software MeV. Compound 2-O-feruloyl-malic acid was not detected in WT and fpgs1, so it is not shown on the heatmap. Abbreviations: f1- fpgs1, cc1- ccoaomt1, f1cc1 - fpgs1ccoaomt1. Number positioned in front of tentatively identified metabolites are the retention time (RT; min) and key mass-to-charge (m/z) ratios.
