# Supplementary material for: Combining loss of function of FOLYLPOLYGLUTAMATE SYNTHETASE1 and CAFFEOYL-COA 3-O-METHYLTRANSFERASE1 for lignin reduction and improved saccharification efficiency in Arabidopsis thaliana
Source: Biotechnol Biofuels. 2019 May 3;12:108. doi: 10.1186/s13068-019-1446-3 (PMC6498598; doi:10.1186/s13068-019-1446-3)
Supplement: Supplementary file 6 — Additional file 6: Table S3. Differentially-accumulated metabolites in 6-week-old stems of WT, fpgs1 (f1), ccoaomt1 (cc1) and fpgs1ccoaomt1 (f1cc1) plants. Selection threshold for significant metabolites (marked with *, up in red, down in blue) is P < 0.05 (Student’s t-test). Metabolite concentration (ug/g fresh weight; sorbitol equivalents). The retention time (RT; min) and key mass-to-charge (m/z) ratios are positioned in front of tentatively identified metabolites. [file 13068_2019_1446_MOESM6_ESM.pptx]

## Slide 1
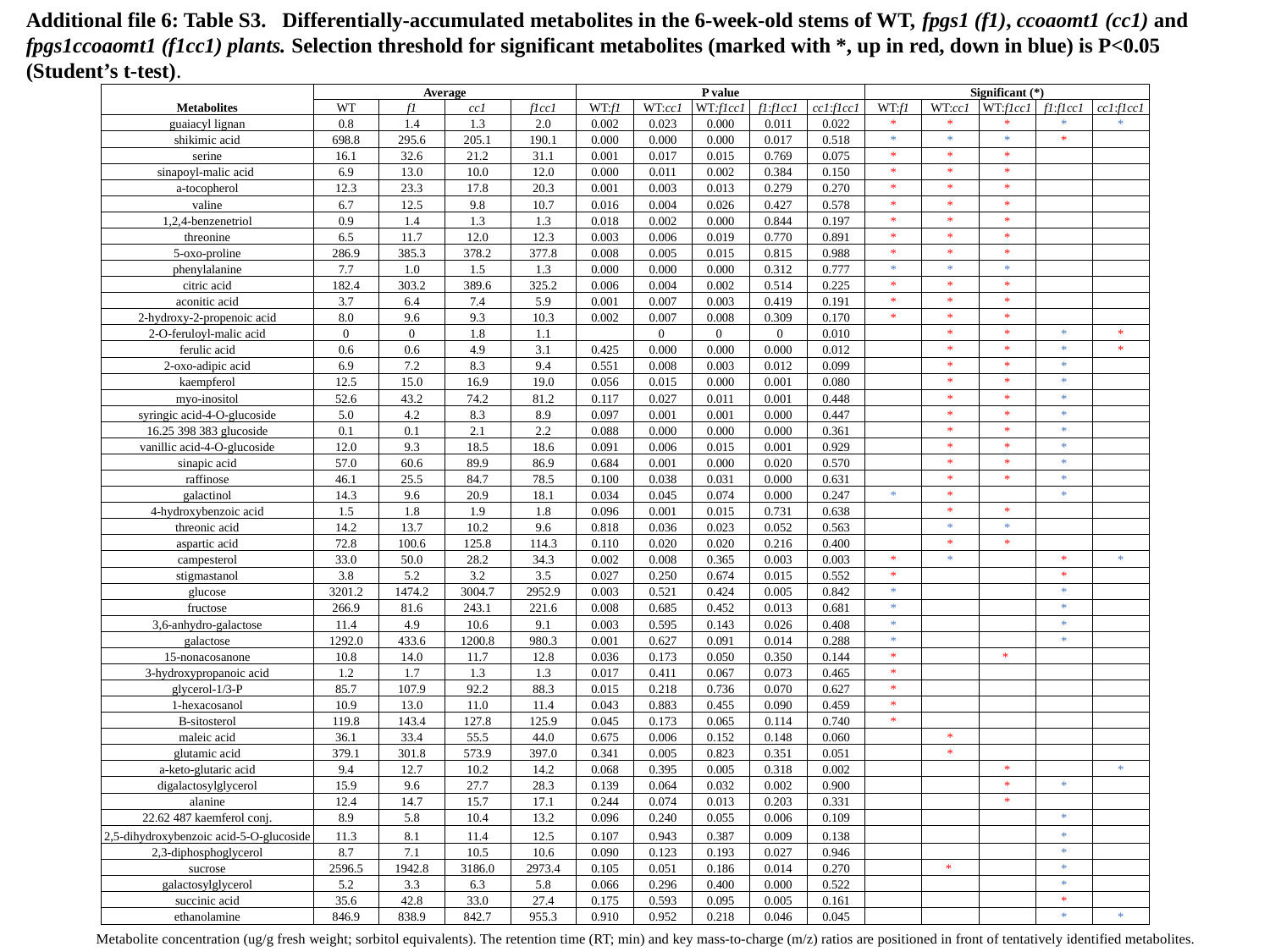

Additional file 6: Table S3. Differentially-accumulated metabolites in the 6-week-old stems of WT, fpgs1 (f1), ccoaomt1 (cc1) and fpgs1ccoaomt1 (f1cc1) plants. Selection threshold for significant metabolites (marked with *, up in red, down in blue) is P<0.05 (Student’s t-test).
| Metabolites | Average | | | | P value | | | | | Significant (\*) | | | | |
| --- | --- | --- | --- | --- | --- | --- | --- | --- | --- | --- | --- | --- | --- | --- |
| | WT | f1 | cc1 | f1cc1 | WT:f1 | WT:cc1 | WT:f1cc1 | f1:f1cc1 | cc1:f1cc1 | WT:f1 | WT:cc1 | WT:f1cc1 | f1:f1cc1 | cc1:f1cc1 |
| guaiacyl lignan | 0.8 | 1.4 | 1.3 | 2.0 | 0.002 | 0.023 | 0.000 | 0.011 | 0.022 | \* | \* | \* | \* | \* |
| shikimic acid | 698.8 | 295.6 | 205.1 | 190.1 | 0.000 | 0.000 | 0.000 | 0.017 | 0.518 | \* | \* | \* | \* | |
| serine | 16.1 | 32.6 | 21.2 | 31.1 | 0.001 | 0.017 | 0.015 | 0.769 | 0.075 | \* | \* | \* | | |
| sinapoyl-malic acid | 6.9 | 13.0 | 10.0 | 12.0 | 0.000 | 0.011 | 0.002 | 0.384 | 0.150 | \* | \* | \* | | |
| a-tocopherol | 12.3 | 23.3 | 17.8 | 20.3 | 0.001 | 0.003 | 0.013 | 0.279 | 0.270 | \* | \* | \* | | |
| valine | 6.7 | 12.5 | 9.8 | 10.7 | 0.016 | 0.004 | 0.026 | 0.427 | 0.578 | \* | \* | \* | | |
| 1,2,4-benzenetriol | 0.9 | 1.4 | 1.3 | 1.3 | 0.018 | 0.002 | 0.000 | 0.844 | 0.197 | \* | \* | \* | | |
| threonine | 6.5 | 11.7 | 12.0 | 12.3 | 0.003 | 0.006 | 0.019 | 0.770 | 0.891 | \* | \* | \* | | |
| 5-oxo-proline | 286.9 | 385.3 | 378.2 | 377.8 | 0.008 | 0.005 | 0.015 | 0.815 | 0.988 | \* | \* | \* | | |
| phenylalanine | 7.7 | 1.0 | 1.5 | 1.3 | 0.000 | 0.000 | 0.000 | 0.312 | 0.777 | \* | \* | \* | | |
| citric acid | 182.4 | 303.2 | 389.6 | 325.2 | 0.006 | 0.004 | 0.002 | 0.514 | 0.225 | \* | \* | \* | | |
| aconitic acid | 3.7 | 6.4 | 7.4 | 5.9 | 0.001 | 0.007 | 0.003 | 0.419 | 0.191 | \* | \* | \* | | |
| 2-hydroxy-2-propenoic acid | 8.0 | 9.6 | 9.3 | 10.3 | 0.002 | 0.007 | 0.008 | 0.309 | 0.170 | \* | \* | \* | | |
| 2-O-feruloyl-malic acid | 0 | 0 | 1.8 | 1.1 | | 0 | 0 | 0 | 0.010 | | \* | \* | \* | \* |
| ferulic acid | 0.6 | 0.6 | 4.9 | 3.1 | 0.425 | 0.000 | 0.000 | 0.000 | 0.012 | | \* | \* | \* | \* |
| 2-oxo-adipic acid | 6.9 | 7.2 | 8.3 | 9.4 | 0.551 | 0.008 | 0.003 | 0.012 | 0.099 | | \* | \* | \* | |
| kaempferol | 12.5 | 15.0 | 16.9 | 19.0 | 0.056 | 0.015 | 0.000 | 0.001 | 0.080 | | \* | \* | \* | |
| myo-inositol | 52.6 | 43.2 | 74.2 | 81.2 | 0.117 | 0.027 | 0.011 | 0.001 | 0.448 | | \* | \* | \* | |
| syringic acid-4-O-glucoside | 5.0 | 4.2 | 8.3 | 8.9 | 0.097 | 0.001 | 0.001 | 0.000 | 0.447 | | \* | \* | \* | |
| 16.25 398 383 glucoside | 0.1 | 0.1 | 2.1 | 2.2 | 0.088 | 0.000 | 0.000 | 0.000 | 0.361 | | \* | \* | \* | |
| vanillic acid-4-O-glucoside | 12.0 | 9.3 | 18.5 | 18.6 | 0.091 | 0.006 | 0.015 | 0.001 | 0.929 | | \* | \* | \* | |
| sinapic acid | 57.0 | 60.6 | 89.9 | 86.9 | 0.684 | 0.001 | 0.000 | 0.020 | 0.570 | | \* | \* | \* | |
| raffinose | 46.1 | 25.5 | 84.7 | 78.5 | 0.100 | 0.038 | 0.031 | 0.000 | 0.631 | | \* | \* | \* | |
| galactinol | 14.3 | 9.6 | 20.9 | 18.1 | 0.034 | 0.045 | 0.074 | 0.000 | 0.247 | \* | \* | | \* | |
| 4-hydroxybenzoic acid | 1.5 | 1.8 | 1.9 | 1.8 | 0.096 | 0.001 | 0.015 | 0.731 | 0.638 | | \* | \* | | |
| threonic acid | 14.2 | 13.7 | 10.2 | 9.6 | 0.818 | 0.036 | 0.023 | 0.052 | 0.563 | | \* | \* | | |
| aspartic acid | 72.8 | 100.6 | 125.8 | 114.3 | 0.110 | 0.020 | 0.020 | 0.216 | 0.400 | | \* | \* | | |
| campesterol | 33.0 | 50.0 | 28.2 | 34.3 | 0.002 | 0.008 | 0.365 | 0.003 | 0.003 | \* | \* | | \* | \* |
| stigmastanol | 3.8 | 5.2 | 3.2 | 3.5 | 0.027 | 0.250 | 0.674 | 0.015 | 0.552 | \* | | | \* | |
| glucose | 3201.2 | 1474.2 | 3004.7 | 2952.9 | 0.003 | 0.521 | 0.424 | 0.005 | 0.842 | \* | | | \* | |
| fructose | 266.9 | 81.6 | 243.1 | 221.6 | 0.008 | 0.685 | 0.452 | 0.013 | 0.681 | \* | | | \* | |
| 3,6-anhydro-galactose | 11.4 | 4.9 | 10.6 | 9.1 | 0.003 | 0.595 | 0.143 | 0.026 | 0.408 | \* | | | \* | |
| galactose | 1292.0 | 433.6 | 1200.8 | 980.3 | 0.001 | 0.627 | 0.091 | 0.014 | 0.288 | \* | | | \* | |
| 15-nonacosanone | 10.8 | 14.0 | 11.7 | 12.8 | 0.036 | 0.173 | 0.050 | 0.350 | 0.144 | \* | | \* | | |
| 3-hydroxypropanoic acid | 1.2 | 1.7 | 1.3 | 1.3 | 0.017 | 0.411 | 0.067 | 0.073 | 0.465 | \* | | | | |
| glycerol-1/3-P | 85.7 | 107.9 | 92.2 | 88.3 | 0.015 | 0.218 | 0.736 | 0.070 | 0.627 | \* | | | | |
| 1-hexacosanol | 10.9 | 13.0 | 11.0 | 11.4 | 0.043 | 0.883 | 0.455 | 0.090 | 0.459 | \* | | | | |
| B-sitosterol | 119.8 | 143.4 | 127.8 | 125.9 | 0.045 | 0.173 | 0.065 | 0.114 | 0.740 | \* | | | | |
| maleic acid | 36.1 | 33.4 | 55.5 | 44.0 | 0.675 | 0.006 | 0.152 | 0.148 | 0.060 | | \* | | | |
| glutamic acid | 379.1 | 301.8 | 573.9 | 397.0 | 0.341 | 0.005 | 0.823 | 0.351 | 0.051 | | \* | | | |
| a-keto-glutaric acid | 9.4 | 12.7 | 10.2 | 14.2 | 0.068 | 0.395 | 0.005 | 0.318 | 0.002 | | | \* | | \* |
| digalactosylglycerol | 15.9 | 9.6 | 27.7 | 28.3 | 0.139 | 0.064 | 0.032 | 0.002 | 0.900 | | | \* | \* | |
| alanine | 12.4 | 14.7 | 15.7 | 17.1 | 0.244 | 0.074 | 0.013 | 0.203 | 0.331 | | | \* | | |
| 22.62 487 kaemferol conj. | 8.9 | 5.8 | 10.4 | 13.2 | 0.096 | 0.240 | 0.055 | 0.006 | 0.109 | | | | \* | |
| 2,5-dihydroxybenzoic acid-5-O-glucoside | 11.3 | 8.1 | 11.4 | 12.5 | 0.107 | 0.943 | 0.387 | 0.009 | 0.138 | | | | \* | |
| 2,3-diphosphoglycerol | 8.7 | 7.1 | 10.5 | 10.6 | 0.090 | 0.123 | 0.193 | 0.027 | 0.946 | | | | \* | |
| sucrose | 2596.5 | 1942.8 | 3186.0 | 2973.4 | 0.105 | 0.051 | 0.186 | 0.014 | 0.270 | | \* | | \* | |
| galactosylglycerol | 5.2 | 3.3 | 6.3 | 5.8 | 0.066 | 0.296 | 0.400 | 0.000 | 0.522 | | | | \* | |
| succinic acid | 35.6 | 42.8 | 33.0 | 27.4 | 0.175 | 0.593 | 0.095 | 0.005 | 0.161 | | | | \* | |
| ethanolamine | 846.9 | 838.9 | 842.7 | 955.3 | 0.910 | 0.952 | 0.218 | 0.046 | 0.045 | | | | \* | \* |
Metabolite concentration (ug/g fresh weight; sorbitol equivalents). The retention time (RT; min) and key mass-to-charge (m/z) ratios are positioned in front of tentatively identified metabolites.
