# Supplementary material for: Combining loss of function of FOLYLPOLYGLUTAMATE SYNTHETASE1 and CAFFEOYL-COA 3-O-METHYLTRANSFERASE1 for lignin reduction and improved saccharification efficiency in Arabidopsis thaliana
Source: Biotechnol Biofuels. 2019 May 3;12:108. doi: 10.1186/s13068-019-1446-3 (PMC6498598; doi:10.1186/s13068-019-1446-3)
Supplement: Supplementary file 7 — Additional file 7: Fig. S4. Presentation of differentially expressed genes in the 6-week-old Arabidopsis stems of fpgs1, ccoaomt1 and fpgs1ccoaomt1 plants compared with WT. Genes with two fold changes in expression levels in fpgs1, ccoaomt1 or fpgs1ccoaomt1, compared with WT, were included in the image. Red - upregulated genes; Blue - downregulated genes. Yellow - each mutants, square - genes in phenylpropanoid/lignin/glucosinolate pathway (At1g80820-AtCCR2; At1g20510-OPCL1; At2g29130-AtLAC2; At1g21110-IGMT3; At1g21120-IGMT2; At4g34050-CCoAOMT1; At1g21130-IGMT4); Triangle - one carbon pathway genes (At5g05980-FPGS1). Lines show the relationship of genes among the mutants. [file 13068_2019_1446_MOESM7_ESM.pptx]

## Slide 1
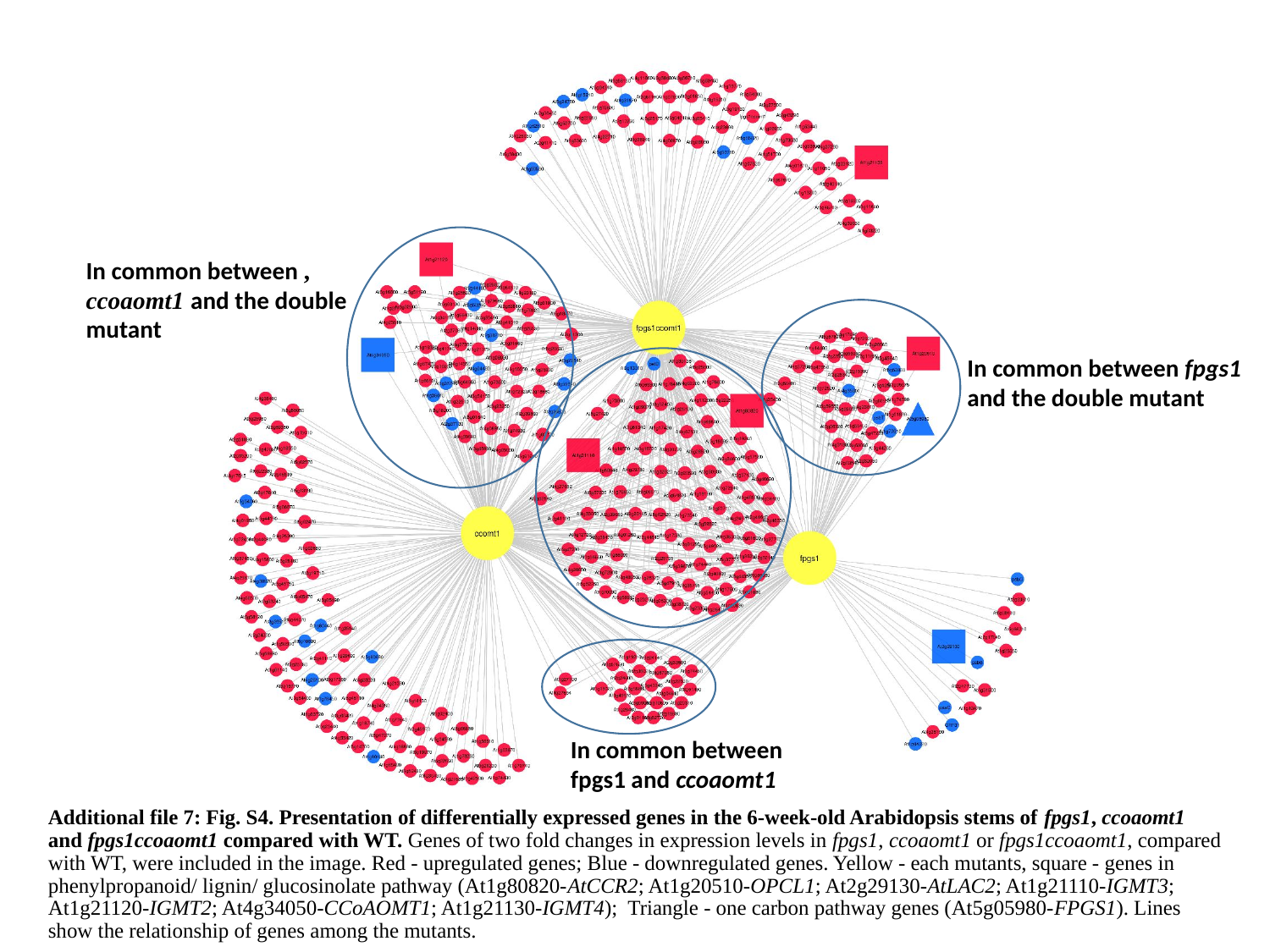

In common between , ccoaomt1 and the double mutant
In common between fpgs1 and the double mutant
In common between fpgs1 and ccoaomt1
Additional file 7: Fig. S4. Presentation of differentially expressed genes in the 6-week-old Arabidopsis stems of fpgs1, ccoaomt1 and fpgs1ccoaomt1 compared with WT. Genes of two fold changes in expression levels in fpgs1, ccoaomt1 or fpgs1ccoaomt1, compared with WT, were included in the image. Red - upregulated genes; Blue - downregulated genes. Yellow - each mutants, square - genes in phenylpropanoid/ lignin/ glucosinolate pathway (At1g80820-AtCCR2; At1g20510-OPCL1; At2g29130-AtLAC2; At1g21110-IGMT3; At1g21120-IGMT2; At4g34050-CCoAOMT1; At1g21130-IGMT4); Triangle - one carbon pathway genes (At5g05980-FPGS1). Lines show the relationship of genes among the mutants.
