# Supplementary material for: Combining loss of function of FOLYLPOLYGLUTAMATE SYNTHETASE1 and CAFFEOYL-COA 3-O-METHYLTRANSFERASE1 for lignin reduction and improved saccharification efficiency in Arabidopsis thaliana
Source: Biotechnol Biofuels. 2019 May 3;12:108. doi: 10.1186/s13068-019-1446-3 (PMC6498598; doi:10.1186/s13068-019-1446-3)
Supplement: Supplementary file 9 — Additional file 9: Fig. S5. Schematic representation of shikimate pathway, aromatic amino acids (AAA) pathway, and lignin pathway association with C1 metabolism. Adapted from the following papers [13, 17, 37, 43, 93–98]. Solid Arrows represent enzymatic reactions together with the name of the enzyme that catalyzes the associated reaction. Empty circles represent the products of the enzymatic reactions along with the products’ names nearby. Dotted arrows represent multiple enzymatic reactions. Red dotted rectangles represent shikimate pathways. Amber dotted rectangles represent aromatic amino acids (AAA) pathways. Green dotted rectangles represent lignin pathways. Purple dotted rectangles represent C1 metabolism. CM: chorismatemutase; PAT: prephenate aminotransferase; ADT: arogenatedehydratase; PAL: phenylalanine ammonia-lyase; C4H: cinnamate-4-hydroxylase; 4CL: 4-coumarateCoAligase; CCR: cinnamoyl-CoA reductase; CAD: cinnamyl alcohol dehydrogenase; C3H: p-Coumarate 3-hydroxylase; HCT: folylpolyglutamate synthase; C3′H: p-coumaroyl quinate/shikimate 3′-hydroxylase, CSE: 5,10-methylene tetrahydrofolate polyglutamates; COMT: caffeic acid O-methyl transferase; CCoAOMT: caffeoyl-CoA O-methyltransferase; F5H: ferulate 5-hydroxylase; CAD: cinnamyl alcohol dehydrogenase; SGT: sinapate:UDP-glucose glucosyltransferase; SMT: sinapoylglucose:malate sinapoyltransferase; ADC: amino deoxychorismate; ADCL: amino deoxychorismate (ADC) lyase; ADCS: amino deoxychorismate (ADC) synthase; pABA: para-aminobenzoic acid; DHPS: dihydropteroate (DHP) synthase; DHFS: dihydrofolate (DHF) synthase; DHF-Glu1: dihydrofolate with one glutamate; DHFR: dihydrofolate (DHF) reductase; THF-Glu1: tetrahydrofolate with one glutamate; FPGS: folylpolyglutamate synthase; THF-Glun: tetrahydrofolate polyglutamates; SHMT: serine hydroxymethyl transferase; CH2-THF-Glun: 5,10-methylene tetrahydrofolate polyglutamates; DHC: 5,10-methylene tetrahydrofolate dehydrogenase/5,10-methenyl THF cyclohydrolase; 10-CHO-THF [file 13068_2019_1446_MOESM9_ESM.pptx]

## Slide 1
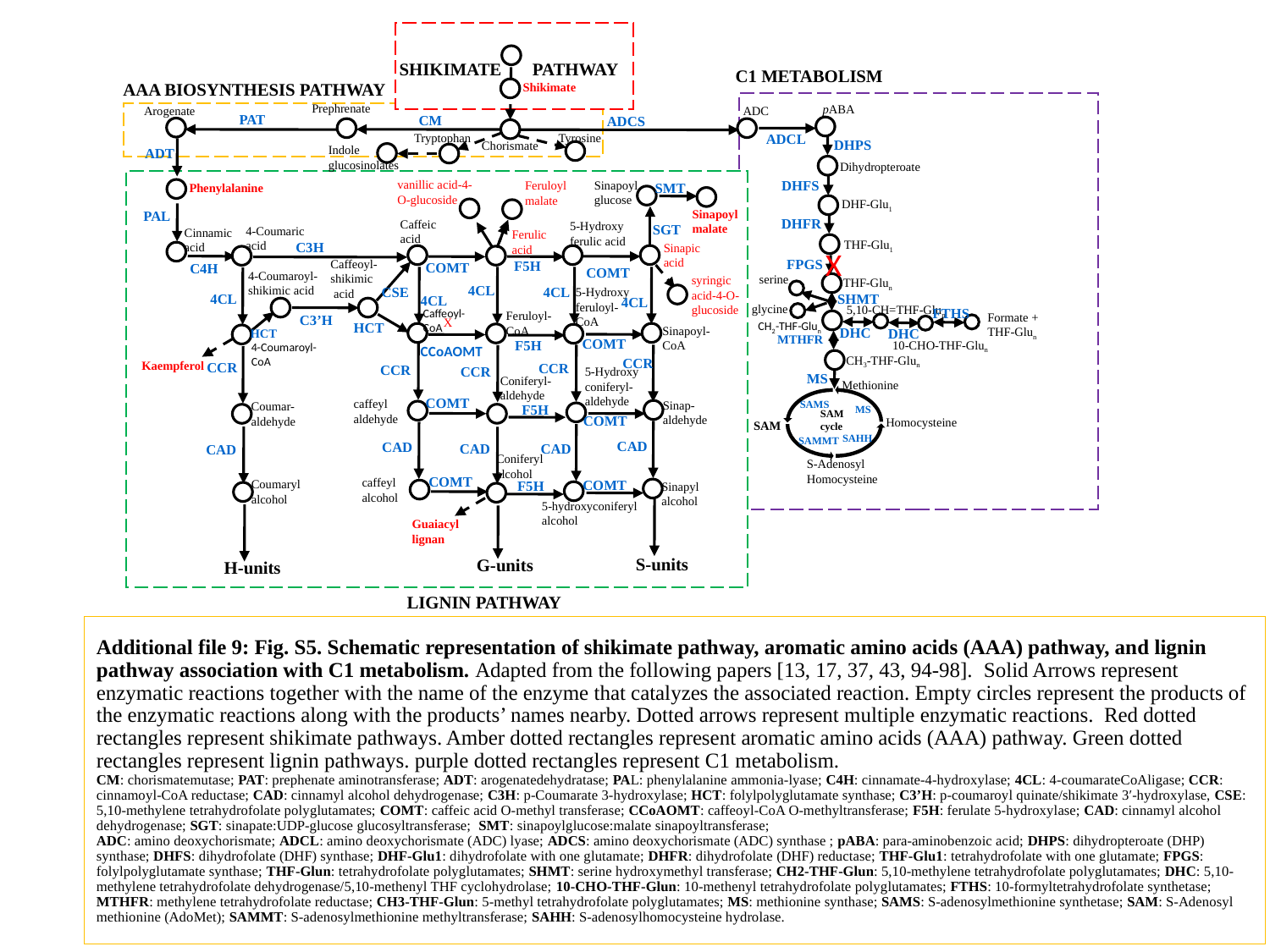

SHIKIMATE PATHWAY
Shikimate
Prephrenate
Arogenate
PAT
CM
ADCS
Tyrosine
Chorismate
Indole glucosinolates
ADT
vanillic acid-4-O-glucoside
Sinapoyl glucose
Feruloyl malate
SMT
Phenylalanine
Sinapoyl malate
PAL
Caffeic
acid
5-Hydroxy
ferulic acid
SGT
4-Coumaric
acid
Cinnamic
acid
Ferulic
acid
C3H
Sinapic
acid
Caffeoyl-
shikimic
 acid
F5H
COMT
C4H
COMT
4-Coumaroyl-
shikimic acid
syringic acid-4-O-glucoside
4CL
CSE
4CL
5-Hydroxy
feruloyl-
CoA
4CL
4CL
4CL
Caffeoyl-
CoA
Feruloyl-
CoA
C3’H
X
HCT
Sinapoyl-
CoA
HCT
COMT
F5H
4-Coumaroyl-
CoA
CCoAOMT
Kaempferol
CCR
CCR
CCR
CCR
5-Hydroxy
coniferyl-
aldehyde
Coniferyl-
aldehyde
COMT
caffeyl
aldehyde
Sinap-
aldehyde
Coumar-
aldehyde
F5H
COMT
CAD
CAD
CAD
CAD
CAD
Coniferyl
alcohol
COMT
caffeyl
alcohol
COMT
Coumaryl
alcohol
F5H
Sinapyl
alcohol
5-hydroxyconiferyl
alcohol
Guaiacyl lignan
S-units
G-units
H-units
CCR
Tryptophan
LIGNIN PATHWAY
C1 METABOLISM
 pABA
ADC
ADCL
DHPS
Dihydropteroate
DHFS
DHF-Glu1
DHFR
THF-Glu1
X
FPGS
serine
glycine
5,10-CH=THF-Glun
FTHS
Formate + THF-Glun
CH2-THF-Glun
DHC
DHC
MTHFR
10-CHO-THF-Glun
CH3-THF-Glun
MS
Methionine
SAMS
MS
Homocysteine
SAM
SAHH
SAMMT
S-Adenosyl
Homocysteine
THF-Glun
SHMT
SAM cycle
AAA BIOSYNTHESIS PATHWAY
Additional file 9: Fig. S5. Schematic representation of shikimate pathway, aromatic amino acids (AAA) pathway, and lignin pathway association with C1 metabolism. Adapted from the following papers [13, 17, 37, 43, 94-98]. Solid Arrows represent enzymatic reactions together with the name of the enzyme that catalyzes the associated reaction. Empty circles represent the products of the enzymatic reactions along with the products’ names nearby. Dotted arrows represent multiple enzymatic reactions. Red dotted rectangles represent shikimate pathways. Amber dotted rectangles represent aromatic amino acids (AAA) pathway. Green dotted rectangles represent lignin pathways. purple dotted rectangles represent C1 metabolism.
CM: chorismatemutase; PAT: prephenate aminotransferase; ADT: arogenatedehydratase; PAL: phenylalanine ammonia-lyase; C4H: cinnamate-4-hydroxylase; 4CL: 4-coumarateCoAligase; CCR: cinnamoyl-CoA reductase; CAD: cinnamyl alcohol dehydrogenase; C3H: p-Coumarate 3-hydroxylase; HCT: folylpolyglutamate synthase; C3’H: p‑coumaroyl quinate/shikimate 3′‑hydroxylase, CSE: 5,10-methylene tetrahydrofolate polyglutamates; COMT: caffeic acid O-methyl transferase; CCoAOMT: caffeoyl-CoA O-methyltransferase; F5H: ferulate 5-hydroxylase; CAD: cinnamyl alcohol dehydrogenase; SGT: sinapate:UDP-glucose glucosyltransferase; SMT: sinapoylglucose:malate sinapoyltransferase;
ADC: amino deoxychorismate; ADCL: amino deoxychorismate (ADC) lyase; ADCS: amino deoxychorismate (ADC) synthase ; pABA: para-aminobenzoic acid; DHPS: dihydropteroate (DHP) synthase; DHFS: dihydrofolate (DHF) synthase; DHF-Glu1: dihydrofolate with one glutamate; DHFR: dihydrofolate (DHF) reductase; THF-Glu1: tetrahydrofolate with one glutamate; FPGS: folylpolyglutamate synthase; THF-Glun: tetrahydrofolate polyglutamates; SHMT: serine hydroxymethyl transferase; CH2-THF-Glun: 5,10-methylene tetrahydrofolate polyglutamates; DHC: 5,10-methylene tetrahydrofolate dehydrogenase/5,10-methenyl THF cyclohydrolase; 10-CHO-THF-Glun: 10-methenyl tetrahydrofolate polyglutamates; FTHS: 10-formyltetrahydrofolate synthetase; MTHFR: methylene tetrahydrofolate reductase; CH3-THF-Glun: 5-methyl tetrahydrofolate polyglutamates; MS: methionine synthase; SAMS: S-adenosylmethionine synthetase; SAM: S-Adenosyl methionine (AdoMet); SAMMT: S-adenosylmethionine methyltransferase; SAHH: S-adenosylhomocysteine hydrolase.
